# Complex spherical fuzzy Aczel Alsina aggregation operators and their application in assessment of electric cars

**DOI:** 10.1016/j.heliyon.2023.e18100

**Published:** 2023-07-07

**Authors:** Abrar Hussain, Kifayat Ullah, Tapan Senapati, Sarbast Moslem

**Affiliations:** a Department of Mathematics, Riphah International University Lahore, Lahore Campus, 5400, Lahore, Pakistan; b School of Mathematics and Statistics, Southwest University, Chongqing, People's Republic of China; c School of Architecture Planning and Environmental Policy, University College of Dublin, D04 V1W8, Belfield, Dublin, Ireland

**Keywords:** Complex spherical fuzzy values, Aggregation operators, Aczel-Alsina operations, Selection process and decision-making process

## Abstract

The multi-criteria decision-making (MCDM) tool is a robust decision-making technique utilized in several fields like networking, risk management, digital analysis, cybercrime investigation, artificial intelligence, waste management enterprises and many other selection criteria. Complex SFS (CSFS) is a new edition of the spherical fuzzy set (SFS) that offers substantial information about any item in terms of amplitude and phase terms in a wider range of real terms. Complex SFS (CSFS) can be an extension of the spherical fuzzy set (SFS). The Aczel-Alsina aggregation tools are more appropriate aggregation operators (AOs), and they are used to conquer the impact of inconsistent and uncertain data. In this paper, we reveal some new approaches based on Aczel-Alsina aggregation tools under consideration of Complex Spherical Fuzzy (CSF) information. These new approaches include the CSF Aczel-Alsina weighted average (CSFAWA) operator, and the CSF Aczel-Alsina ordered weighted average (CSFOWA) operator. In addition to this, we also introduce a list of novel techniques by making use of the theory of Aczel-Alsina aggregation tools such as CSF Aczel-Alsina weighted geometric (CSFAWG) and CSF Aczel-Alsina ordered weighted geometric (CSFOWG) operators. To demonstrate the resilience and efficacy of the approaches that have been mentioned, we will examine a few exceptional examples and remarkable properties of the methodology that we have devised. In addition, a characterization is provided for an approach to the MCDM issue using the CPF information system. We use the example of electric automobiles as a case study to illustrate the uniformity and dependability of the methodology that we have established. This example was chosen because of the high cost of fuel and the present economic challenges that are being encountered by families in the middle class. An empirical case study is also constructed to determine an electric car that is desirable based on the techniques that we have proposed. To evaluate the correctness and superiority of the established strategies, we compare the outcomes of previously used techniques with the AOs currently being provided.

## 1 Introduction

Problems in making decisions are widespread throughout a wide variety of industries, including marketing, finance, urban mobility, and technology, amongst others. It has been a common misconception that all information on alternative accessibility is stored as discrete integers. Therefore it is necessary to manage the ambiguity and imprecision that come with data when dealing with circumstances in the actual world. When it comes to selection, there are three possible responses or attitudes: yes, no, and rejection. Zadeh [1] presented the mathematical foundations of fuzzy sets, often known as FSs, and their implications in a wide variety of domains, including the theory of decision-making, management sciences, engineering, computer science, and many more. The FS theory needs to have several significant flaws. One of these is the fact that Zadeh only took into account an object's membership value (MV) in FSs. In response to this drawback, Atanassov [2] proposed the unique idea of intuitionistic FSs (IFSs). This useful tool captures two distinct degrees; a membership value denoted by MV with the same meaning as above and a non-membership value denoted by (NMV). They are required to meet the requirement that at any moment their sum should be less than or equal to 1. After establishing an IFS, certain models focused on broadening the scope of their operations by either relaxing the limitations they imposed or incorporating new capabilities. Yager [3,4] proposed, the idea of pythagorean FS (PyFS) after generalizing an IFSs by relaxing the requirement on MV ″α″ and NMV ″β″ to $\alpha^{2}+\beta^{2}$. The theory of q-rung orthopair FS (q-ROFS) presented by the Yager [5] with relax the condition of MV and NMV such as $\alpha^{q}+\beta^{q},q\in Z^{+}$. Picture fuzzy set (PFS), which is also more extensive than IFS, was introduced by Cuong [[6], [7], [8]]. To achieve this, they added three degrees of an element to the PFS: MD α, abstinence value (AV) or neutral value (NV) ν, and NMD β; each of these degrees satisfies the joint requirement α+ν+β at any point. PFSs can handle human views of the following types: yes, abstain, no, and rejection. In 2019, Mahmood et al. [9] explored the theory of the PFSs known as spherical FS (SFS). Using the condition $\alpha^{2}+\nu^{2}+\beta^{2}$, this model expanded the space of MD α, AD ν, and NMD β in the range [0,1]. Ullah et al. [10] extended the theory SFSs to evaluate a suitable policy for investment. The readers are directed to the following sources for more information on SFSs and their applications read in Refs. [[11], [12], [13]].

All the above-discussed environments cannot deal with periodic systems and two-dimensional scenarios. To deal with such a situation, Ramot et al. [14,15] gave the theory of complex FSs (CFSs) by expanding the range of membership value (MV) to the unit circle in the complex plan. Alkouri and Salleh [16] extended the concepts of CFSs with MV and non-membership value (NMV) to a unit disk in a complex plane and further generalization of CFSs seen in Refs. [[17], [18], [19], [20], [21]].

Numerous research scientists in the field of decision-making, pattern recognition and medical identification etc., utilize the theory of aggregation tools. Calvo et al. [22] produced theoretic concepts about weighted average and weighted geometric operators. Xu [23], Xu and Yager [24] explored the concepts of aggregation tools and developed a list of new approaches to overcome uncertain and dubious data about human opinions. Azeem et al. [25] derived the idea of CIFSs and derived a particular collection of AOs based on Einstein aggregation tools. Kumar and Chen [26] gave a list of new AOs to overcome the shortcoming of existing approaches by utilizing the advanced theory of linguistic IF information and established a multi-criteria group decision-making (MCGDM) approach to solve real-life issues [[60], [61]]. Khan et al. [27] proposed some new methodologies by combining two different concepts of Schweizer-Sklar and Power aggregation tools under the system of IF circumstances. Rahim et al. [28] described the significance of the trigonometric function and proposed a list of new approaches based on PyF information to solve an MCDM problem. Fuping et al. [29] generalized the theory of FSs in the form of hesitant probabilistic FSs, and pythagorean triangular FSs and introduced certain new approaches based on PyF information to solve an MCDM technique. Application of green supplier management based on the MCDM approach and AOs of the q-ROF information by utilizing the concepts of hypersoft ordered aggregation tools are given by Khan et al. [30]. Zhang et al. [31] proposed new approaches to SF information based on Dombi power Heronian mean and also studied an application of a MAGDM technique. Riaz and Farid [32] developed a structure of new AOs to overcome dubious and complicated information in a computational and intelligent system based on SF circumstances. An application of vendor management classification and a list of new approaches based on CPFSs was established by Hussain et al. [33]. Hussain et al. [34] determined an application of the tourism industry and gave some new approaches to CIFSs based on Hamy mean tools. Ullah et al. [35] established aggregation tools to overcome vagueness and impression information during the decision-making process under the CPyF environment.

Recently, a list of serval AOs of Aczel-Alsina aggregation tools has been developed by numerous research scientists. Farahbod and Eftekhari [36] studied the nature of different triangular norms and classified more effective ones; after investigation and evaluation, they concluded that Aczel-Alsina produced more appropriate results than others. Aczel-Alsina aggregation tools have become a hot research topic these days. Some mathematical aggregation approaches using the theory of Aczel-Alsina tools based on IFSs were developed by Senapati et al. [37]. They also explored the concepts of Aczel-Alsina triangular norms in different fuzzy circumstances and provided some appropriate methodologies [[[38], [39], [40], [41]]. Hussain et al. [42,43] produced some new approaches using the basic operations of Aczel-Alsina aggregation tools based on PyF information to solve an application under the MCDM technique. Jin et al. [44] anticipated certain approaches based on complex PFSs and established an experimental case to solve real-life problems. Naeem et al. [45] presented appropriate PFS methodologies based on PFSs to overcome dubious and vague information. Some certain and flexible AOs of SFSs were developed by Riaz et al. [46]. Mahmood et al. [47] proposed a list of new approaches and gave some particular properties of Complex IFSs based on Aczel-Alsina aggregation tools. Hussain et al. [48] illustrated the theory of Heronian mean models under intuitionistic fuzzy circumstances. Ahmmad et al. [49] derived the theory of intuitionistic fuzzy rough sets and introduced new methodologies to solve experimental cases under medical diagnosis. Mahmood and Ali [50] modified the theory of complex IFSs to define interrelationships between different arguments during the aggregation process by utilizing newly developed approaches. Some particular AOs using Aczel-Alsina aggregation tools developed by Ali and Naeem [51].

All the above-examined existing methodologies cannot overcome insufficient vague and complicated information during the aggregation process. A CSFS contains extensive information about any object, with three terms, each of which has two aspects in the form of amplitude and phase terms. By inspiring the significance of CSFS, we explored the theory of CSFSs to cope with awkward and imprecision information. Some prominent characteristics and properties based on CSFSs are present here. There are many generalizations of triangular norms, such as Frank aggregation tools, Dombi aggregation tools, operations of the Einstein aggregation model, Hamacher aggregation tools and many other extensions of the triangular norms. However, Aczel-Alsina aggregation tools are more appropriate AOs than above discussed extension of the triangular norms. Aczel-Alsina aggregation tools provide a smooth approximation and accurate results during the aggregation process. However, numerous researchers explored the theory of Aczel-Alsina aggregation tools under different fuzzy domains. But there needs to be work presented by any scholar under the system of CPF information. Sometimes experts need help with decision-making due to insufficient information about any object. To overcome such challenges, the theory of CSF information is more beneficial by inspiring the theoretical structure of the CSFSs, which is an extended concept of the PFS and SFS. Basic operational laws of Aczel-Alsina aggregation tools are characterized under the system of CSF information. To see the advantages and effectiveness of Aczel-Alsina aggregation tools under the system of the CSFS, we proposed some appropriate methodologies, including CSFAWA, CSFAOWA, CSFAWG and CSFAOWG operators. A brief discussion about certain properties of currently proposed methodologies is also explained.

Moreover, we applied our invented approaches to solve an MCDM approach. We illustrate an experimental case of electric motor cars to examine the validity and reliability of our discussed aggregation approaches. Comparative analysis, advantages and limitations of our current research work are also described in detail.

The structure of this manuscript is organized as follows: in Section 1, we examined the history of our current research work and existing aggregation mathematical tools. In Section 2, some notions of CSFSs and their basic fundamental operations. In Section 3, an extension of triangular norms in the form of Aczel-Alsina mathematical tools is also present here. In Section 4, some appropriate AOs of CSFSs are based on Aczel-Alsina aggregation tools, including CSFAWA and CSFAOWA operators. Section 5 also explored concepts of geometric aggregation tools in the form of CSFAWG and CSFAOWG operators. In Section 6, an MCDM technique also studies to choose more suitable electric cars based on our invented methodologies. In Section 7, a comparison between the results of the existing approaches and the results of current methodologies is also present. Section 8 concludes our research work, including solicitations and advantages of current work and expresses our future directions.

## 2 Preliminaries

In this section, a brief discussion about SFSs, CSFSs and their related basic operations are also present. We studied comparison methods of CSF information based on score values and accuracy values. This symbol M indicates the universal set throughout this article. (Table 1), covered all symbols with their meanings, which are utilized in this article.

Definition 1 [10] A SFS ∁ is given by:

∁={ǻ,(α(ǻ),ν(ǻ),β(ǻ))|ǻ∈M}

where α(ǻ),ν(ǻ),β(ǻ)∈M→[0,1], represents the MV, AV and NMV of the SFS. A SFS must satisfy the following axiom:

$$0\leq \alpha^{2}\left(ǻ\right)+\nu^{2}\left(ǻ\right)+\beta^{2}\left(ǻ\right)\leq 1$$

**Table 1:** Symbols and their meanings.

Symbols	Meanings	symbols	Meanings
α	Membership value	ℶ	Scalar multiple
β	Non-membership value	Ⱥ	Parametric value
ν	Abstinence value	ῠ	CSF value
M	Universal set	Ϗ	Weight vectors
ǻ	Element belongs to the Universal set	E	Decision matrix
Ϡ	Refusal value	∁	Set
∅	Phase term of MV	ω	Phase term of NMV
φ	Phase term of AV	S	Score Value

The refusal value of the SFS is denoted by $Ϡ\left(ǻ\right)={\left(1-\left(\alpha^{2}\left(ǻ\right)+\nu^{2}\left(ǻ\right)+\beta^{2}\left(ǻ\right)\right)\right)}^{\frac{1}{2}}$.

Definition 2 [14] A CFS ∁ is given by:

∁={(ǻ,Ϋ(ǻ))|,ǻ∈M}

where $Ϋ=\alpha \left(ǻ\right)e^{2i\pi \emptyset \left(ǻ\right)}$ such that α(ǻ)∈[0,1] and ∅(ǻ)∈[0,1] denote the MV of the amplitude and phase terms of CFS respectively. A CFS satisfy the following conditions:

0≤α(ǻ)≤1,and0≤∅(ǻ)≤1

Definition 3 [52] A CSFS is given as:

∁={ǻ,(Ϋ(ǻ),у(ǻ),щ(ǻ))|,ǻ∈M}

where $Ϋ\left(ǻ\right)=\alpha \left(ǻ\right)e^{2i\pi \emptyset \left(ǻ\right)},у\left(ǻ\right)=\nu \left(ǻ\right)e^{2i\pi \varphi \left(ǻ\right)}$ and $щ\left(ǻ\right)=,\beta \left(ǻ\right)e^{2i\pi \omega \left(ǻ\right)}$ represent the MV, AV and NMV respectively. Thus, amplitude terms of MV, AV and NMV are denoted by α(ǻ)∈[0,1],ν(ǻ)∈[0,1] and β(ǻ)∈[0,1] respectively. Similarly, phase terms of MV, AV and NMV are denoted by ∅(ǻ)∈[0,1],φ(ǻ)∈[0,1] and ω(ǻ)∈[0,1] respectively. A CSFS satisfies the following axioms:

$$0\leq \alpha^{2}\left(ǻ\right)+\nu^{2}\left(ǻ\right)+\beta^{2}\left(ǻ\right)\leq 1,and\;0\leq \emptyset^{2}\left(ǻ\right)+\varphi^{2}\left(ǻ\right)+\omega^{2}\left(ǻ\right)\leq 1$$

The refusal value of a CSFS is denoted by the $Ϡ\left(ǻ\right)=\left(\begin{array}{c} {\left(1-\left(\alpha^{2}\left(ǻ\right)+\nu^{2}\left(ǻ\right)+\beta^{2}\left(ǻ\right)\right)\right)}^{\frac{1}{2}}\\ e^{2\pi \left({\left(1-\left(\emptyset^{2}\left(ǻ\right)+\varphi^{2}\left(ǻ\right)+\omega^{2}\left(ǻ\right)\right)\right)}^{\frac{1}{2}}\right)} \end{array}\right)$. Further, a CSF value (CSFV) is denoted by $ῠ=\left\{\alpha \left(ǻ\right)e^{2i\pi \emptyset \left(ǻ\right)},\nu \left(ǻ\right)e^{2i\pi \varphi \left(ǻ\right)},\beta \left(ǻ\right)e^{2i\pi \omega \left(ǻ\right)}\right\}$.

Definition 4 [53] Suppose $ῠ=\left\{\alpha \left(ǻ\right)e^{2i\pi \emptyset \left(ǻ\right)},\nu \left(ǻ\right)e^{2i\pi \varphi \left(ǻ\right)},\beta \left(ǻ\right)e^{2i\pi \omega \left(ǻ\right)}\right\},{ῠ}_{1}=\left\{\alpha_{1}\left(ǻ\right)e^{2i\pi \emptyset_{1}\left(ǻ\right)},\nu_{1}\left(ǻ\right)e^{2i\pi \varphi_{1}\left(ǻ\right)},\beta_{1}\left(ǻ\right)e^{2i\pi \omega_{1}\left(ǻ\right)}\right\}$ and ${ῠ}_{2}=\left\{\alpha_{2}\left(ǻ\right)e^{2i\pi \emptyset_{2}\left(ǻ\right)},\nu_{2}\left(ǻ\right)e^{2i\pi \varphi_{2}\left(ǻ\right)},\beta_{2}\left(ǻ\right)e^{2i\pi \omega_{2}\left(ǻ\right)}\right\}$ are three CSFVs on M and ℶ>0. Then we have:

$$a)\;{ῠ}_{1}{⊕ῠ}_{2}=\left(\begin{array}{c} \sqrt{\alpha_{1}^{2}\left(ǻ\right)+\alpha_{2}^{2}\left(ǻ\right)-\alpha_{1}^{2}\left(ǻ\right)\alpha_{2}^{2}\left(ǻ\right)}e^{2i\pi \left(\sqrt{\emptyset_{1}^{2}\left(ǻ\right)+\emptyset_{2}^{2}\left(ǻ\right)-\emptyset_{1}^{2}\left(ǻ\right)\emptyset_{2}^{2}\left(ǻ\right)}\right)},\\ \nu_{1}\left(ǻ\right).\nu_{2}\left(ǻ\right)e^{2i\pi \left(\varphi_{1}\left(ǻ\right).\varphi_{2}\left(ǻ\right)\right)},\\ \beta_{1}\left(ǻ\right).\beta_{2}\left(ǻ\right)e^{2i\pi \left(\omega_{1}\left(ǻ\right).\;\omega_{2}\left(ǻ\right)\right)} \end{array}\right)$$

$$b)\;{ῠ}_{1}{⊕ῠ}_{2}=\left(\begin{array}{c} \alpha_{1}\left(ǻ\right).\alpha_{2}\left(ǻ\right)e^{2i\pi \left(\emptyset_{1}\left(ǻ\right).\emptyset_{2}\left(ǻ\right)\right)},\\ \sqrt{\nu_{1}^{2}\left(ǻ\right)+\nu_{2}^{2}\left(ǻ\right)-\nu_{1}^{2}\left(ǻ\right)\nu_{2}^{2}\left(ǻ\right)}e^{2i\pi \left(\sqrt{\varphi_{1}^{2}\left(ǻ\right)+\varphi_{2}^{2}\left(ǻ\right)-\varphi_{1}^{2}\left(ǻ\right)\varphi_{2}^{2}\left(ǻ\right)}\right)},\\ \sqrt{\beta_{1}^{2}\left(ǻ\right)+\beta_{2}^{2}\left(ǻ\right)-\beta_{1}^{2}\left(ǻ\right)\beta_{2}^{2}\left(ǻ\right)}e^{2i\pi \left(\sqrt{\omega_{1}^{2}\left(ǻ\right)+\omega_{2}^{2}\left(ǻ\right)-\omega_{1}^{2}\left(ǻ\right)\omega_{2}^{2}\left(ǻ\right)}\right)} \end{array}\right)$$

$$c)\;ℶῠ=\left(\begin{array}{c} \sqrt{1-{\left(1-\alpha^{2}\left(ǻ\right)\right)}^{ℶ}}e^{2i\pi \left(\sqrt{1-{\left(1-\emptyset^{2}\left(ǻ\right)\right)}^{ℶ}}\right)},\\ \nu^{ℶ}\left(ǻ\right)e^{2i\pi \varphi^{ℶ}\left(ǻ\right)},\beta^{ℶ}\left(ǻ\right)e^{2i\pi \omega^{ℶ}\left(ǻ\right)} \end{array}\right)$$

$$d)\;{ῠ}^{ℶ}=\left(\begin{array}{c} \alpha^{ℶ}\left(ǻ\right)e^{2i\pi \emptyset^{ℶ}\left(ǻ\right)},\\ \sqrt{1-{\left(1-\nu^{2}\left(ǻ\right)\right)}^{ℶ}}e^{2i\pi \left(\sqrt{1-{\left(1-\varphi^{2}\left(ǻ\right)\right)}^{ℶ}}\right)},\\ \sqrt{1-{\left(1-\beta^{2}\left(ǻ\right)\right)}^{ℶ}}e^{2i\pi \left(\sqrt{1-{\left(1-\omega^{2}\left(ǻ\right)\right)}^{ℶ}}\right)} \end{array}\right)$$

$$e)\;\bar{ῠ}=\left\{\beta \left(ǻ\right)e^{2i\pi \omega \left(ǻ\right)},\nu \left(ǻ\right)e^{2i\pi \varphi \left(ǻ\right)},\alpha \left(ǻ\right)e^{2i\pi \emptyset \left(ǻ\right)}\right\}$$

Definition 5 [53] For $ῠ=\left\{\alpha \left(ǻ\right)e^{2i\pi \emptyset \left(ǻ\right)},\nu \left(ǻ\right)e^{2i\pi \varphi \left(ǻ\right)},\beta \left(ǻ\right)e^{2i\pi \omega \left(ǻ\right)}\right\}$ be a CSFV and the score value of CSFV is defined as follows:

$$S\left(ῠ\right)=\frac{1}{6}\left((2+\alpha^{2}\left(ǻ\right)-\nu^{2}\left(ǻ\right)-\beta^{2}\left(ǻ\right)\right)+\left(2+\emptyset^{2}\left(ǻ\right)-\varphi^{2}\left(ǻ\right)-\omega^{2}\left(ǻ\right)\right)$$

where S(ῠ)∈[0,1].

## 3 Aczel-Alsina operations

We provided a detailed discussion about the basic operational laws of the Aczel-Alsina aggregation models based on CSF information.

Definition 6 Let $ῠ=\left(\alpha e^{2i\pi \left(\emptyset \right)},\nu e^{2i\pi \left(\varphi \right)},\beta e^{2i\pi \left(\omega \right)}\right),{ῠ}_{1}=\left(\alpha_{1}e^{2i\pi \left(\emptyset_{1}\right)},\upsilon_{1}e^{2i\pi \left(\varphi_{1}\right)},\beta_{1}e^{2i\pi \left(\omega_{1}\right)}\right)$ and ${ῠ}_{2}=\left(\alpha_{2}e^{2i\pi \left(\emptyset_{2}\right)},\upsilon_{2}e^{2i\pi \left(\varphi_{2}\right)},\beta_{2}e^{2i\pi \left(\omega_{2}\right)}\right)$ be any three CSFVs, and Λ>0,Ⱥ≥1. Then necessary operational laws of CSFVs based on Aczel-Alsina aggregation tools are given by:

$${ῠ}_{1}⨁{ῠ}_{2}=\left(\begin{array}{c} \sqrt{1-{e^{-\left({\left(-\log\left(1-\alpha_{1}^{2}\right)\right)}^{Ⱥ}+{\left(\left(-\log\left(1-\alpha_{2}^{2}\right)\right)\right)}^{Ⱥ}\right)}}^{\frac{1}{Ⱥ}}}e^{2i\pi \left(\sqrt{1-{e^{-\left({\left(-\log\left(1-\emptyset_{1}^{2}\right)\right)}^{Ⱥ}+{\left(\left(-\log\left(1-\emptyset_{2}^{2}\right)\right)\right)}^{Ⱥ}\right)}}^{\frac{1}{Ⱥ}}}\right)},\\ {e^{-\left({\left(-\log\left(\nu_{1}\right)\right)}^{Ⱥ}+{\left(\left(-\log\left(\nu_{2}\right)\right)\right)}^{Ⱥ}\right)}}^{\frac{1}{Ⱥ}}e^{2i\pi \left({e^{-\left({\left(-\log\left(\varphi_{1}\right)\right)}^{Ⱥ}+{\left(\left(-\log\left(\varphi_{2}\right)\right)\right)}^{Ⱥ}\right)}}^{\frac{1}{Ⱥ}}\right)},\\ {e^{-\left({\left(-\log\left(\beta_{1}\right)\right)}^{Ⱥ}+{\left(\left(-\log\left(\beta_{2}\right)\right)\right)}^{Ⱥ}\right)}}^{\frac{1}{Ⱥ}}e^{2i\pi \left({e^{-\left({\left(-\log\left(\omega_{1}\right)\right)}^{Ⱥ}+{\left(\left(-\log\left(\omega_{2}\right)\right)\right)}^{Ⱥ}\right)}}^{\frac{1}{Ⱥ}}\right)} \end{array}\right)$$

$${ῠ}_{1}⨂{ῠ}_{2}=\left(\begin{array}{c} {e^{-\left({\left(-\log\left(\alpha_{1}\right)\right)}^{Ⱥ}+{\left(\left(-\log\left(\alpha_{2}\right)\right)\right)}^{Ⱥ}\right)}}^{\frac{1}{Ⱥ}}e^{2i\pi \left({e^{-\left({\left(-\log\left(\emptyset_{1}\right)\right)}^{Ⱥ}+{\left(\left(-\log\left(\emptyset_{2}\right)\right)\right)}^{Ⱥ}\right)}}^{\frac{1}{Ⱥ}}\right)},\\ \sqrt{1-{e^{-\left({\left(-\log\left(1-\nu_{1}^{2}\right)\right)}^{Ⱥ}+{\left(\left(-\log\left(1-\nu_{2}^{2}\right)\right)\right)}^{Ⱥ}\right)}}^{\frac{1}{Ⱥ}}}e^{2i\pi \left(\sqrt{1-{e^{-\left({\left(-\log\left(1-\varphi_{1}^{2}\right)\right)}^{Ⱥ}+{\left(\left(-\log\left(1-\varphi_{2}^{2}\right)\right)\right)}^{Ⱥ}\right)}}^{\frac{1}{Ⱥ}}}\right)},\\ \sqrt{1-{e^{-\left({\left(-\log\left(1-\beta_{1}^{2}\right)\right)}^{Ⱥ}+{\left(\left(-\log\left(1-\beta_{2}^{2}\right)\right)\right)}^{Ⱥ}\right)}}^{\frac{1}{Ⱥ}}}e^{2i\pi \left(\sqrt{1-{e^{-\left({\left(-\log\left(1-\omega_{1}^{2}\right)\right)}^{Ⱥ}+{\left(\left(-\log\left(1-\omega_{2}^{2}\right)\right)\right)}^{Ⱥ}\right)}}^{\frac{1}{Ⱥ}}}\right)} \end{array}\right)$$

$$\Lambda ῠ=\left(\begin{array}{c} \sqrt{1-{e^{-\left(\Lambda {\left(-\log\left(1-\alpha^{2}\right)\right)}^{Ⱥ}\right)}}^{\frac{1}{Ⱥ}}}e^{2i\pi \left(\sqrt{1-{e^{-\left({\Lambda \left(-\log\left(1-\emptyset^{2}\right)\right)}^{Ⱥ}\right)}}^{\frac{1}{Ⱥ}}}\right)},\\ {e^{-\left({\Lambda \left(-\log\left(\nu \right)\right)}^{Ⱥ}\right)}}^{\frac{1}{Ⱥ}}e^{2i\pi \left({e^{-\left(\Lambda {\left(-\log\left(\varphi \right)\right)}^{Ⱥ}\right)}}^{\frac{1}{Ⱥ}}\right)},\\ {e^{-\left(\Lambda {\left(-\log\left(\beta \right)\right)}^{Ⱥ}\right)}}^{\frac{1}{Ⱥ}}e^{2i\pi \left({e^{-\left({\Lambda \left(-\log\left(\omega \right)\right)}^{Ⱥ}\right)}}^{\frac{1}{Ⱥ}}\right)} \end{array}\right)$$

$${ῠ}^{\Lambda }=\left(\begin{array}{c} {e^{-\left({\Lambda \left(-\log\left(\alpha \right)\right)}^{Ⱥ}\right)}}^{\frac{1}{Ⱥ}}e^{2i\pi \left({e^{-\left(\Lambda {\left(-\log\left(\emptyset \right)\right)}^{Ⱥ}\right)}}^{\frac{1}{Ⱥ}}\right)},\\ \sqrt{1-{e^{-\left(\Lambda {\left(-\log\left(1-\nu^{2}\right)\right)}^{Ⱥ}\right)}}^{\frac{1}{Ⱥ}}}e^{2i\pi \left(\sqrt{1-{e^{-\left({\Lambda \left(-\log\left(1-\varphi^{2}\right)\right)}^{Ⱥ}\right)}}^{\frac{1}{Ⱥ}}}\right)},\\ \sqrt{1-{e^{-\left(\Lambda {\left(-\log\left(1-\beta^{2}\right)\right)}^{Ⱥ}\right)}}^{\frac{1}{Ⱥ}}}e^{2i\pi \left(\sqrt{1-{e^{-\left({\Lambda \left(-\log\left(1-\omega^{2}\right)\right)}^{Ⱥ}\right)}}^{\frac{1}{Ⱥ}}}\right)} \end{array}\right)$$

## 4 Complex spherical fuzzy Aczel-Alsina aggregation operators

In this portion, we provided a list of new approaches of the CSFSs in the form of CSFAAWA and CSFAAOWA operators with certain properties of our invented approaches. In this article, the associated weight vector of ${ῠ}_{ɤ}$ is represented by ${Ϗ}_{ɤ}=\left({Ϗ}_{1},{Ϗ}_{2},\ldots ,{Ϗ}_{ƞ}\right),ɤ=1,2,3,\ldots ,ƞ$ such that Ϗ∈[0,1] and $\sum_{ϼ=1}^{ƞ}{Ϗ}_{ɤ}$, utilized these weight vectors throughout this article.

Definition 7 Let ${ῠ}_{ɤ}=\left(\alpha_{ɤ}e^{2i\pi \left(\emptyset_{ɤ}\right)},\nu_{ɤ}e^{2i\pi \left(\varphi_{ɤ}\right)},\beta_{ɤ}e^{2i\pi \left(\omega_{ɤ}\right)}\right),ɤ=1,2,3,\ldots ,ƞ$ be the set of SFNs. Then CSFAAWA operator is particularized as:

$$CSFAAWA\left({ῠ}_{1},{ῠ}_{2},{ῠ}_{3},\ldots ,{ῠ}_{ƞ}\right)=\overset{ƞ}{\underset{ɤ=1}{⨁}}{Ϗ}_{ɤ}{ῠ}_{ɤ}={Ϗ}_{1}{ῠ}_{1}⊕{Ϗ}_{2}{ῠ}_{2}⊕,\ldots ,⊕{Ϗ}_{ƞ}{ῠ}_{ƞ}$$

Theorem 1 *Let*${ῠ}_{ɤ}=\left(\alpha_{ɤ}e^{2i\pi \left(\emptyset_{ɤ}\right)},\nu_{ɤ}e^{2i\pi \left(\varphi_{ɤ}\right)},\beta_{ɤ}e^{2i\pi \left(\omega_{ɤ}\right)}\right),ɤ=1,2,3,\ldots ,ƞ$*be the set of SFNs*. *Then the integrated values of the CSFAAWA operator still a CSFV*, *so we have the*:

$$CSFAAWA\left({ῠ}_{1},{ῠ}_{2},{ῠ}_{3},\ldots ,{ῠ}_{ƞ}\right)=\left(\begin{array}{c} \sqrt{1-{e^{-\left(\sum_{ɤ=1}^{ƞ}\left({Ϗ}_{ɤ}{\left(-\log\left(1-\alpha_{ɤ}^{2}\right)\right)}^{Ⱥ}\right)\right)}}^{\frac{1}{Ⱥ}}}e^{2i\pi \left(\sqrt{1-{e^{-\left(\sum_{ɤ=1}^{ƞ}\left({Ϗ}_{ɤ}{\left(-\log\left(1-\emptyset_{ɤ}^{2}\right)\right)}^{Ⱥ}\right)\right)}}^{\frac{1}{Ⱥ}}}\right)},\\ e^{-{\left(\sum_{ɤ=1}^{ƞ}\left({{Ϗ}_{ɤ}\left(-\log\left(\nu_{ɤ}\right)\right)}^{Ⱥ}\right)\right)}^{\frac{1}{Ⱥ}}}e^{2i\pi \left({e^{-\left(\sum_{ɤ=1}^{ƞ}\left({{Ϗ}_{ɤ}\left(-\log\left(\varphi_{ɤ}\right)\right)}^{Ⱥ}\right)\right)}}^{\frac{1}{Ⱥ}}\right)},\\ {e^{-\left(\sum_{ɤ=1}^{ƞ}{Ϗ}_{ɤ}{\left(-\log\left(\beta_{ɤ}\right)\right)}^{Ⱥ}\right)}}^{\frac{1}{Ⱥ}}e^{2i\pi \left({e^{-\left(\sum_{ɤ=1}^{ƞ}{{Ϗ}_{ɤ}\left(-\log\left(\omega_{ɤ}\right)\right)}^{Ⱥ}\right)}}^{\frac{1}{Ⱥ}}\right)} \end{array}\right)$$

Proof: To prove the above theorem, we use the mathematical induction technique for ƞ=2, so we have:

$${Ϗ}_{1}{ῠ}_{1}=\left(\begin{array}{c} \sqrt{1-{e^{-\left({Ϗ}_{1}{\left(-\log\left(1-\alpha_{1}^{2}\right)\right)}^{Ⱥ}\right)}}^{\frac{1}{Ⱥ}}}e^{2i\pi \left(\sqrt{1-{e^{-\left({{Ϗ}_{1}\left(-\log\left(1-\emptyset_{1}^{2}\right)\right)}^{Ⱥ}\right)}}^{\frac{1}{Ⱥ}}}\right)},\\ {e^{-\left({{Ϗ}_{1}\left(-\log\left(\nu_{1}\right)\right)}^{Ⱥ}\right)}}^{\frac{1}{Ⱥ}}e^{2i\pi \left({e^{-\left({Ϗ}_{1}{\left(-\log\left(\varphi_{1}\right)\right)}^{Ⱥ}\right)}}^{\frac{1}{Ⱥ}}\right)},\\ {e^{-\left({Ϗ}_{1}{\left(-\log\left(\beta \right)\right)}^{Ⱥ}\right)}}^{\frac{1}{Ⱥ}}e^{2i\pi \left({e^{-\left({{Ϗ}_{1}\left(-\log\left(\omega_{1}\right)\right)}^{Ⱥ}\right)}}^{\frac{1}{Ⱥ}}\right)} \end{array}\right)$$

$${Ϗ}_{2}{ῠ}_{2}=\left(\begin{array}{c} \sqrt{1-{e^{-\left({Ϗ}_{2}{\left(-\log\left(1-\alpha_{2}^{2}\right)\right)}^{Ⱥ}\right)}}^{\frac{1}{Ⱥ}}}e^{2i\pi \left(\sqrt{1-{e^{-\left({{Ϗ}_{2}\left(-\log\left(1-\emptyset_{2}^{2}\right)\right)}^{Ⱥ}\right)}}^{\frac{1}{Ⱥ}}}\right)},\\ {e^{-\left({{Ϗ}_{2}\left(-\log\left(\nu_{2}\right)\right)}^{Ⱥ}\right)}}^{\frac{1}{Ⱥ}}e^{2i\pi \left({e^{-\left({Ϗ}_{2}{\left(-\log\left(\varphi_{2}\right)\right)}^{Ⱥ}\right)}}^{\frac{1}{Ⱥ}}\right)},\\ {e^{-\left({Ϗ}_{2}{\left(-\log\left(\beta_{2}\right)\right)}^{Ⱥ}\right)}}^{\frac{1}{Ⱥ}}e^{2i\pi \left({e^{-\left({{Ϗ}_{2}\left(-\log\left(\omega_{2}\right)\right)}^{Ⱥ}\right)}}^{\frac{1}{Ⱥ}}\right)} \end{array}\right)$$

$${Ϗ}_{1}{ῠ}_{1}⊕{Ϗ}_{2}{ῠ}_{2}=\left(\begin{array}{c} \sqrt{1-{e^{-\left(\left({Ϗ}_{1}{\left(-\log\left(1-\alpha_{1}^{2}\right)\right)}^{Ⱥ}\right)+\left({Ϗ}_{2}{\left(-\log\left(1-\alpha_{2}^{2}\right)\right)}^{Ⱥ}\right)\right)}}^{\frac{1}{Ⱥ}}}e^{2i\pi \left(\sqrt{1-{e^{-\left(\left({Ϗ}_{1}{\left(-\log\left(1-\emptyset_{1}^{2}\right)\right)}^{Ⱥ}\right)+\left({Ϗ}_{2}{\left(-\log\left(1-\emptyset_{2}^{2}\right)\right)}^{Ⱥ}\right)\right)}}^{\frac{1}{Ⱥ}}}\right)},\\ e^{-{\left(\left({{Ϗ}_{1}\left(-\log\left(\nu_{1}\right)\right)}^{Ⱥ}\right)+\left({{Ϗ}_{2}\left(-\log\left(\nu_{2}\right)\right)}^{Ⱥ}\right)\right)}^{\frac{1}{Ⱥ}}}e^{2i\pi \left({e^{-\left(\left({{Ϗ}_{1}\left(-\log\left(\varphi_{1}\right)\right)}^{Ⱥ}\right)+\left({{Ϗ}_{2}\left(-\log\left(\varphi_{2}\right)\right)}^{Ⱥ}\right)\right)}}^{\frac{1}{Ⱥ}}\right)},\\ {e^{-\left({Ϗ}_{ɤ}{\left(-\log\left(\beta_{1}\right)\right)}^{Ⱥ}+{Ϗ}_{ɤ}{\left(\left(-\log\left(\beta_{2}\right)\right)\right)}^{Ⱥ}\right)}}^{\frac{1}{Ⱥ}}e^{2i\pi \left({e^{-\left({{Ϗ}_{ɤ}\left(-\log\left(\omega_{1}\right)\right)}^{Ⱥ}+{{Ϗ}_{ɤ}\left(\left(-\log\left(\omega_{2}\right)\right)\right)}^{Ⱥ}\right)}}^{\frac{1}{Ⱥ}}\right)} \end{array}\right)$$

$$=\left(\begin{array}{c} \sqrt{1-{e^{-\left(\sum_{ɤ=1}^{2}\left({Ϗ}_{ɤ}{\left(-\log\left(1-\alpha_{ɤ}^{2}\right)\right)}^{Ⱥ}\right)\right)}}^{\frac{1}{Ⱥ}}}e^{2i\pi \left(\sqrt{1-{e^{-\left(\sum_{ɤ=1}^{2}\left({Ϗ}_{ɤ}{\left(-\log\left(1-\emptyset_{ɤ}^{2}\right)\right)}^{Ⱥ}\right)\right)}}^{\frac{1}{Ⱥ}}}\right)},\\ e^{-{\left(\sum_{ɤ=1}^{2}\left({{Ϗ}_{ɤ}\left(-\log\left(\nu_{ɤ}\right)\right)}^{Ⱥ}\right)\right)}^{\frac{1}{Ⱥ}}}e^{2i\pi \left({e^{-\left(\sum_{ɤ=1}^{2}\left({{Ϗ}_{ɤ}\left(-\log\left(\varphi_{ɤ}\right)\right)}^{Ⱥ}\right)\right)}}^{\frac{1}{Ⱥ}}\right)},\\ {e^{-\left(\sum_{ɤ=1}^{2}{Ϗ}_{ɤ}{\left(-\log\left(\beta_{ɤ}\right)\right)}^{Ⱥ}\right)}}^{\frac{1}{Ⱥ}}e^{2i\pi \left({e^{-\left(\sum_{ɤ=1}^{2}{{Ϗ}_{ɤ}\left(-\log\left(\omega_{ɤ}\right)\right)}^{Ⱥ}\right)}}^{\frac{1}{Ⱥ}}\right)} \end{array}\right)$$

thus, this is true for ƞ=2.

Now for ƞ=k we have the following equation

$$=\left(\begin{array}{c} \sqrt{1-{e^{-\left(\sum_{ɤ=1}^{k}\left({Ϗ}_{ɤ}{\left(-\log\left(1-\alpha_{ɤ}^{2}\right)\right)}^{Ⱥ}\right)\right)}}^{\frac{1}{Ⱥ}}}e^{2i\pi \left(\sqrt{1-{e^{-\left(\sum_{ɤ=1}^{k}\left({Ϗ}_{ɤ}{\left(-\log\left(1-\emptyset_{ɤ}^{2}\right)\right)}^{Ⱥ}\right)\right)}}^{\frac{1}{Ⱥ}}}\right)},\\ e^{-{\left(\sum_{ɤ=1}^{k}\left({{Ϗ}_{ɤ}\left(-\log\left(\nu_{ɤ}\right)\right)}^{Ⱥ}\right)\right)}^{\frac{1}{Ⱥ}}}e^{2i\pi \left({e^{-\left(\sum_{ɤ=1}^{k}\left({{Ϗ}_{ɤ}\left(-\log\left(\varphi_{ɤ}\right)\right)}^{Ⱥ}\right)\right)}}^{\frac{1}{Ⱥ}}\right)},\\ {e^{-\left(\sum_{ɤ=1}^{k}{Ϗ}_{ɤ}{\left(-\log\left(\beta_{ɤ}\right)\right)}^{Ⱥ}\right)}}^{\frac{1}{Ⱥ}}e^{2i\pi \left({e^{-\left(\sum_{ɤ=1}^{k}{{Ϗ}_{ɤ}\left(-\log\left(\omega_{ɤ}\right)\right)}^{Ⱥ}\right)}}^{\frac{1}{Ⱥ}}\right)} \end{array}\right)$$

now we have to show that the above equation is true for ƞ=k+1.

$$CSFAAWA\left({ῠ}_{1},{ῠ}_{2},{ῠ}_{3},\ldots ,{ῠ}_{k},{ῠ}_{k+1}\right)=\overset{k+1}{\underset{ɤ=1}{⨁}}{Ϗ}_{ɤ}{ῠ}_{ɤ}=\overset{k}{\underset{ɤ=1}{⨁}}{Ϗ}_{ɤ}{ῠ}_{ɤ}⊕{Ϗ}_{k+1}{ῠ}_{k+1}$$

$$=\left(\begin{array}{c} \sqrt{1-{e^{-\left(\sum_{ɤ=1}^{k}\left({Ϗ}_{ɤ}{\left(-\log\left(1-\alpha_{ɤ}^{2}\right)\right)}^{Ⱥ}\right)\right)}}^{\frac{1}{Ⱥ}}}e^{2i\pi \left(\sqrt{1-{e^{-\left(\sum_{ɤ=1}^{k}\left({Ϗ}_{ɤ}{\left(-\log\left(1-\emptyset_{ɤ}^{2}\right)\right)}^{Ⱥ}\right)\right)}}^{\frac{1}{Ⱥ}}}\right)},\\ e^{-{\left(\sum_{ɤ=1}^{k}\left({{Ϗ}_{ɤ}\left(-\log\left(\nu_{ɤ}\right)\right)}^{Ⱥ}\right)\right)}^{\frac{1}{Ⱥ}}}e^{2i\pi \left({e^{-\left(\sum_{ɤ=1}^{k}\left({{Ϗ}_{ɤ}\left(-\log\left(\varphi_{ɤ}\right)\right)}^{Ⱥ}\right)\right)}}^{\frac{1}{Ⱥ}}\right)},\\ {e^{-\left(\sum_{ɤ=1}^{k}{{Ϗ}_{ɤ}\left(-\log\left(\beta_{ɤ}\right)\right)}^{Ⱥ}\right)}}^{\frac{1}{Ⱥ}}e^{2i\pi \left({e^{-\left(\sum_{ɤ=1}^{k}{Ϗ}_{ɤ}{\left(-\log\left(\omega_{ɤ}\right)\right)}^{Ⱥ}\right)}}^{\frac{1}{Ⱥ}}\right)} \end{array}\right)⊕\left(\begin{array}{c} \sqrt{1-{e^{-\left({Ϗ}_{k+1}{\left(-\log\left(1-\alpha_{k+1}^{2}\right)\right)}^{Ⱥ}\right)}}^{\frac{1}{Ⱥ}}}e^{2i\pi \left(\sqrt{1-{e^{-\left({Ϗ}_{k+1}{\left(-\log\left(1-\emptyset_{k+1}^{2}\right)\right)}^{Ⱥ}\right)}}^{\frac{1}{Ⱥ}}}\right)},\\ e^{-{\left(\left({{Ϗ}_{k+1}\left(-\log\left(\nu_{k+1}\right)\right)}^{Ⱥ}\right)\right)}^{\frac{1}{Ⱥ}}}e^{2i\pi \left({e^{-\left(\left({{Ϗ}_{k+1}\left(-\log\left(\varphi_{k+1}\right)\right)}^{Ⱥ}\right)\right)}}^{\frac{1}{Ⱥ}}\right)},\\ {e^{-\left({{Ϗ}_{ɤ}\left(-\log\left(\beta_{ɤ}\right)\right)}^{Ⱥ}\right)}}^{\frac{1}{Ⱥ}}e^{2i\pi \left({e^{-\left({Ϗ}_{ɤ}{\left(-\log\left(\omega_{ɤ}\right)\right)}^{Ⱥ}\right)}}^{\frac{1}{Ⱥ}}\right)} \end{array}\right)$$

$$CSFAAWA\left({ῠ}_{1},{ῠ}_{2},{ῠ}_{3},\ldots ,{ῠ}_{k},{ῠ}_{k+1}\right)=\left(\begin{array}{c} \sqrt{1-{e^{-\left(\sum_{ɤ=1}^{k+1}\left({Ϗ}_{ɤ}{\left(-\log\left(1-\alpha_{ɤ}^{2}\right)\right)}^{Ⱥ}\right)\right)}}^{\frac{1}{Ⱥ}}}e^{2i\pi \left(\sqrt{1-{e^{-\left(\sum_{ɤ=1}^{k+1}\left({Ϗ}_{ɤ}{\left(-\log\left(1-\emptyset_{ɤ}^{2}\right)\right)}^{Ⱥ}\right)\right)}}^{\frac{1}{Ⱥ}}}\right)},\\ e^{-{\left(\sum_{ɤ=1}^{k+1}\left({{Ϗ}_{ɤ}\left(-\log\left(\nu_{ɤ}\right)\right)}^{Ⱥ}\right)\right)}^{\frac{1}{Ⱥ}}}e^{2i\pi \left({e^{-\left(\sum_{ɤ=1}^{k+1}\left({{Ϗ}_{ɤ}\left(-\log\left(\varphi_{ɤ}\right)\right)}^{Ⱥ}\right)\right)}}^{\frac{1}{Ⱥ}}\right)},\\ {e^{-\left(\sum_{ɤ=1}^{k+1}{Ϗ}_{ɤ}{\left(-\log\left(\beta_{ɤ}\right)\right)}^{Ⱥ}\right)}}^{\frac{1}{Ⱥ}}e^{2i\pi \left({e^{-\left(\sum_{ɤ=1}^{k+1}{{Ϗ}_{ɤ}\left(-\log\left(\omega_{ɤ}\right)\right)}^{Ⱥ}\right)}}^{\frac{1}{Ⱥ}}\right)} \end{array}\right)$$

so, after examining we conclude that the above equation is true for ƞ=k+1.

Therefore, we have:

$$CSFAAWA\left({ῠ}_{1},{ῠ}_{2},{ῠ}_{3},\ldots ,{ῠ}_{ƞ}\right)=\left(\begin{array}{c} \sqrt{1-{e^{-\left(\sum_{ɤ=1}^{ƞ}\left({Ϗ}_{ɤ}{\left(-\log\left(1-\alpha_{ɤ}^{2}\right)\right)}^{Ⱥ}\right)\right)}}^{\frac{1}{Ⱥ}}}e^{2i\pi \left(\sqrt{1-{e^{-\left(\sum_{ɤ=1}^{ƞ}\left({Ϗ}_{ɤ}{\left(-\log\left(1-\emptyset_{ɤ}^{2}\right)\right)}^{Ⱥ}\right)\right)}}^{\frac{1}{Ⱥ}}}\right)},\\ e^{-{\left(\sum_{ɤ=1}^{ƞ}\left({{Ϗ}_{ɤ}\left(-\log\left(\nu_{ɤ}\right)\right)}^{Ⱥ}\right)\right)}^{\frac{1}{Ⱥ}}}e^{2i\pi \left({e^{-\left(\sum_{ɤ=1}^{ƞ}\left({{Ϗ}_{ɤ}\left(-\log\left(\varphi_{ɤ}\right)\right)}^{Ⱥ}\right)\right)}}^{\frac{1}{Ⱥ}}\right)},\\ {e^{-\left(\sum_{ɤ=1}^{ƞ}{Ϗ}_{ɤ}{\left(-\log\left(\beta_{ɤ}\right)\right)}^{Ⱥ}\right)}}^{\frac{1}{Ⱥ}}e^{2i\pi \left({e^{-\left(\sum_{ɤ=1}^{ƞ}{{Ϗ}_{ɤ}\left(-\log\left(\omega_{ɤ}\right)\right)}^{Ⱥ}\right)}}^{\frac{1}{Ⱥ}}\right)} \end{array}\right)$$

Example 1 Consider four CSFVs are ${ῠ}_{1}=\left(0.34e^{2i\pi \left(0.15\right)},0.61e^{2i\pi \left(0.22\right)},0.37e^{2i\pi \left(0.15\right)}\right),{ῠ}_{2}=\left(0.18e^{2i\pi \left(0.37\right)},0.27e^{2i\pi \left(0.28\right)},0.41e^{2i\pi \left(0.81\right)}\right),{ῠ}_{3}=\left(0.63e^{2i\pi \left(0.45\right)},0.16e^{2i\pi \left(0.67\right)},0.31e^{2i\pi \left(0.43\right)}\right)$ and ${ῠ}_{4}=\left(0.36e^{2i\pi \left(0.29\right)},0.49e^{2i\pi \left(0.24\right)},0.45e^{2i\pi \left(0.39\right)}\right)$ and let Ϗ=(0.23,0.27,0.30,0.20) be the weight vector of the given CSFVs. Then the solution of given aggregated values obtained by the CSFAAWA operators is given as suppose Ⱥ=3.

Solution:

$$CSFAAWA\left({ῠ}_{1},{ῠ}_{2},{ῠ}_{3},\ldots ,{ῠ}_{ƞ}\right)=\left(\begin{array}{c} \sqrt{1-{e^{-\left(\sum_{ɤ=1}^{ƞ}\left({Ϗ}_{ɤ}{\left(-\log\left(1-\alpha_{ɤ}^{2}\right)\right)}^{Ⱥ}\right)\right)}}^{\frac{1}{Ⱥ}}}e^{2i\pi \left(\sqrt{1-{e^{-\left(\sum_{ɤ=1}^{ƞ}\left({Ϗ}_{ɤ}{\left(-\log\left(1-\emptyset_{ɤ}^{2}\right)\right)}^{Ⱥ}\right)\right)}}^{\frac{1}{Ⱥ}}}\right)},\\ e^{-{\left(\sum_{ɤ=1}^{ƞ}\left({{Ϗ}_{ɤ}\left(-\log\left(\nu_{ɤ}\right)\right)}^{Ⱥ}\right)\right)}^{\frac{1}{Ⱥ}}}e^{2i\pi \left({e^{-\left(\sum_{ɤ=1}^{ƞ}\left({{Ϗ}_{ɤ}\left(-\log\left(\varphi_{ɤ}\right)\right)}^{Ⱥ}\right)\right)}}^{\frac{1}{Ⱥ}}\right)},\\ {e^{-\left(\sum_{ɤ=1}^{ƞ}{Ϗ}_{ɤ}{\left(-\log\left(\beta_{ɤ}\right)\right)}^{Ⱥ}\right)}}^{\frac{1}{Ⱥ}}e^{2i\pi \left({e^{-\left(\sum_{ɤ=1}^{ƞ}{{Ϗ}_{ɤ}\left(-\log\left(\omega_{ɤ}\right)\right)}^{Ⱥ}\right)}}^{\frac{1}{Ⱥ}}\right)} \end{array}\right)$$

$$CSFAAWA\left({ῠ}_{1},{ῠ}_{2},{ῠ}_{3},{ῠ}_{4}\right)=\left(\begin{array}{c} \sqrt{1-e^{-{\left(\left(\begin{array}{c} \left(0.23\right){\left(-\log\left(1-{\left(0.34\right)}^{2}\right)\right)}^{3}+\left(0.27\right){\left(-\log\left(1-{\left(0.18\right)}^{2}\right)\right)}^{3}+\\ \left(0.30\right){\left(-\log\left(1-{\left(0.63\right)}^{2}\right)\right)}^{3}+\left(0.20\right){\left(-\log\left(1-{\left(0.36\right)}^{2}\right)\right)}^{3} \end{array}\right)\right)}^{\frac{1}{3}}}}\\ e^{2\pi i\left(\sqrt{1-e^{-{\left(\left(\begin{array}{c} \left(0.23\right){\left(-\log\left(1-{\left(0.15\right)}^{2}\right)\right)}^{3}+\left(0.27\right){\left(-\log\left(1-{\left(0.37\right)}^{2}\right)\right)}^{3}+\\ \left(0.30\right){\left(-\log\left(1-{\left(0.45\right)}^{2}\right)\right)}^{3}+\left(0.20\right){\left(-\log\left(1-{\left(0.29\right)}^{2}\right)\right)}^{3} \end{array}\right)\right)}^{\frac{1}{3}}}}\right)},\\ e^{-{\left(\left(\begin{array}{c} {\left(0.23\right)\left(-\log\left(0.61\right)\right)}^{3}+{\left(0.27\right)\left(-\log\left(0.27\right)\right)}^{3}+\\ {\left(0.30\right)\left(-\log\left(0.16\right)\right)}^{3}+{\left(0.20\right)\left(-\log\left(0.49\right)\right)}^{3} \end{array}\right)\right)}^{\frac{1}{3}}}\\ e^{2\pi i\left(e^{-{\left(\left(\begin{array}{c} {\left(0.23\right)\left(-\log\left(0.22\right)\right)}^{3}+{\left(0.27\right)\left(-\log\left(0.28\right)\right)}^{3}+\\ {\left(0.30\right)\left(-\log\left(0.67\right)\right)}^{3}+{\left(0.20\right)\left(-\log\left(0.24\right)\right)}^{3} \end{array}\right)\right)}^{\frac{1}{3}}}\right)},\\ e^{-{\left(\left(\begin{array}{c} {\left(0.23\right)\left(-\log\left(0.37\right)\right)}^{3}+{\left(0.27\right)\left(-\log\left(0.41\right)\right)}^{3}+\\ {\left(0.30\right)\left(-\log\left(0.31\right)\right)}^{3}+{\left(0.20\right)\left(-\log\left(0.45\right)\right)}^{3} \end{array}\right)\right)}^{\frac{1}{3}}}\\ e^{2\pi i\left(e^{-{\left(\left(\begin{array}{c} {\left(0.23\right)\left(-\log\left(0.15\right)\right)}^{3}+{\left(0.27\right)\left(-\log\left(0.81\right)\right)}^{3}+\\ {\left(0.30\right)\left(-\log\left(0.43\right)\right)}^{3}+{\left(0.20\right)\left(-\log\left(0.39\right)\right)}^{3} \end{array}\right)\right)}^{\frac{1}{3}}}\right)} \end{array}\right)$$

$$=\left(0.3712e^{2i\pi \left(0.2628\right)},0.5524e^{2i\pi \left(0.5809\right)},0.6476e^{2i\pi \left(0.5829\right)}\right)$$

Theorem 2 *If all*${ῠ}_{ɤ}=\left(\alpha_{ɤ}e^{2i\pi \left(\emptyset_{ɤ}\right)},\nu_{ɤ}e^{2i\pi \left(\varphi_{ɤ}\right)},\beta_{ɤ}e^{2i\pi \left(\omega_{ɤ}\right)}\right)$*be the set of all equal CSFVs*, *that is*${ῠ}_{ɤ}=ῠ,\forall ,ɤ\text{.}$*Then*, *we have*:

$$CSFAAWA\left({ῠ}_{1},{ῠ}_{2},{ῠ}_{3},\ldots ,{ῠ}_{ƞ}\right)=ῠ$$

*Proof*: *Given that*${ῠ}_{ɤ}=\left(\alpha_{ɤ}e^{2i\pi \left(\emptyset_{ɤ}\right)},\nu_{ɤ}e^{2i\pi \left(\varphi_{ɤ}\right)},\beta_{ɤ}e^{2i\pi \left(\omega_{ɤ}\right)}\right)$*by equation*, *we get the equation*:

$$CSFAAWA\left({ῠ}_{1},{ῠ}_{2},{ῠ}_{3},\ldots ,{ῠ}_{ƞ}\right)=\left(\begin{array}{c} \sqrt{1-{e^{-\left(\sum_{ɤ=1}^{ƞ}\left({Ϗ}_{ɤ}{\left(-\log\left(1-\alpha_{ɤ}^{2}\right)\right)}^{Ⱥ}\right)\right)}}^{\frac{1}{Ⱥ}}}e^{2i\pi \left(\sqrt{1-{e^{-\left(\sum_{ɤ=1}^{ƞ}\left({Ϗ}_{ɤ}{\left(-\log\left(1-\emptyset_{ɤ}^{2}\right)\right)}^{Ⱥ}\right)\right)}}^{\frac{1}{Ⱥ}}}\right)},\\ e^{-{\left(\sum_{ɤ=1}^{ƞ}\left({{Ϗ}_{ɤ}\left(-\log\left(\nu_{ɤ}\right)\right)}^{Ⱥ}\right)\right)}^{\frac{1}{Ⱥ}}}e^{2i\pi \left({e^{-\left(\sum_{ɤ=1}^{ƞ}\left({{Ϗ}_{ɤ}\left(-log\left(\varphi_{ɤ}\right)\right)}^{Ⱥ}\right)\right)}}^{\frac{1}{Ⱥ}}\right)},\\ {e^{-\left(\sum_{ɤ=1}^{ƞ}{Ϗ}_{ɤ}{\left(-\log\left(\beta_{ɤ}\right)\right)}^{Ⱥ}\right)}}^{\frac{1}{Ⱥ}}e^{2i\pi \left({e^{-\left(\sum_{ɤ=1}^{ƞ}{{Ϗ}_{ɤ}\left(-\log\left(\omega_{ɤ}\right)\right)}^{Ⱥ}\right)}}^{\frac{1}{Ⱥ}}\right)} \end{array}\right)$$

$$=\left(\begin{array}{c} \sqrt{1-{e^{-\left(\left({Ϗ}_{ɤ}{\left(-\log\left(1-\alpha^{2}\right)\right)}^{Ⱥ}\right)\right)}}^{\frac{1}{Ⱥ}}}e^{2i\pi \left(\sqrt{1-{e^{-\left(\left({Ϗ}_{ɤ}{\left(-\log\left(1-\emptyset^{2}\right)\right)}^{Ⱥ}\right)\right)}}^{\frac{1}{Ⱥ}}}\right)},\\ e^{-{\left(\left({{Ϗ}_{ɤ}\left(-\log\left(\nu \right)\right)}^{Ⱥ}\right)\right)}^{\frac{1}{Ⱥ}}}e^{2i\pi \left({e^{-\left(\left({{Ϗ}_{ɤ}\left(-\log\left(\varphi \right)\right)}^{Ⱥ}\right)\right)}}^{\frac{1}{Ⱥ}}\right)},\\ {e^{-\left(\left({Ϗ}_{ɤ}{\left(-\log\left(\beta \right)\right)}^{Ⱥ}\right)\right)}}^{\frac{1}{Ⱥ}}e^{2i\pi \left({e^{-\left(\left({{Ϗ}_{ɤ}\left(-\log\left(\omega \right)\right)}^{Ⱥ}\right)\right)}}^{\frac{1}{Ⱥ}}\right)} \end{array}\right)$$

$$=\left(\alpha e^{2i\pi \left(\emptyset \right)},\nu e^{2i\pi \left(\varphi \right)},\beta e^{2i\pi \left(\omega \right)}\right)=ῠ$$

Theorem 3 *Let*${ῠ}_{ɤ}=\left(\alpha_{ɤ}e^{2i\pi \left(\emptyset_{ɤ}\right)},\nu_{ɤ}e^{2i\pi \left(\varphi_{ɤ}\right)},\beta_{ɤ}e^{2i\pi \left(\omega_{ɤ}\right)}\right)$*be the set of CSFVs*, *and if*${ῠ}^{-}=\min\left(\left({ῠ}_{1},{ῠ}_{2},{ῠ}_{3},\ldots ,{ῠ}_{ƞ}\right)\right)$*and*${ῠ}^{+}=\max\left({ῠ}_{1},{ῠ}_{2},{ῠ}_{3},\ldots ,{ῠ}_{ƞ}\right)$. *Then we get*:

$${ῠ}^{-}\leq CSFAAWA\left({ῠ}_{1},{ῠ}_{2},{ῠ}_{3},\ldots ,{ῠ}_{ƞ}\right)\leq {ῠ}^{+}$$

Proof: Let ${ῠ}_{ɤ}=\left(\alpha_{ɤ}e^{2i\pi \left(\emptyset_{ɤ}\right)},\nu_{ɤ}e^{2i\pi \left(\varphi_{ɤ}\right)},\beta_{ɤ}e^{2i\pi \left(\omega_{ɤ}\right)}\right)$ be the set of CSFVs, Let

$${ῠ}^{-}=\min\left({ῠ}_{1},{ῠ}_{2},{ῠ}_{3},\ldots ,{ῠ}_{ƞ}\right)=\left(\alpha_{ɤ}^{-}e^{2i\pi \emptyset_{ɤ}^{-}},\nu_{ɤ}^{-}e^{2i\pi \varphi_{ɤ}^{-}},\beta_{ɤ}^{-}e^{2i\pi \omega_{ɤ}^{-}}\right)$$

and

$${ῠ}^{+}=\max\left({ῠ}_{1},{ῠ}_{2},{ῠ}_{3},\ldots ,{ῠ}_{ƞ}\right)=\left(\alpha_{ɤ}^{+}e^{2i\pi \emptyset_{ɤ}^{+}},\nu_{ɤ}^{+}e^{2i\pi \varphi_{ɤ}^{+}},\beta_{ɤ}^{+}e^{2i\pi \omega_{ɤ}^{+}}\right)$$.

Since

$$\alpha^{-}e^{2i\pi \emptyset^{-}}=\min_{ɤ}\left\{\alpha_{ɤ}e^{2i\pi \emptyset_{ɤ}}\right\},\nu^{-}e^{2i\pi \varphi^{-}}=\max_{ɤ}\left\{\nu_{ɤ}e^{2i\pi \left(\varphi_{ɤ}\right)}\right\},\beta^{-}e^{2i\pi \omega_{ɤ}^{-}}=\max_{ɤ}\left\{\beta_{ɤ}e^{2i\pi \left(\omega_{ɤ}\right)}\right\}$$

and

$$\alpha^{+}e^{2i\pi \left(\emptyset_{ɤ}^{+}\right)}=\max_{ɤ}\left\{\alpha_{ɤ}e^{2i\pi \left(\emptyset_{ɤ}\right)}\right\},\nu^{+}e^{2i\pi \left(\varphi_{ɤ}^{+}\right)}=\min_{ɤ}\left\{\nu_{ɤ}e^{2i\pi \left(\varphi_{ɤ}\right)}\right\},\beta^{+}e^{2i\pi \left(\omega_{ɤ}^{+}\right)}=\min_{ɤ}\left\{\beta_{ɤ}e^{2i\pi \left(\omega_{ɤ}\right)}\right\}$$.

Hence, we have the subsequent inequalities:

$$\sqrt{1-{e^{-\left(\sum_{ɤ=1}^{ƞ}\left({Ϗ}_{ɤ}{\left(-\log\left(1-{\left(\alpha_{ῠ}^{-}\right)}^{2}\right)\right)}^{Ⱥ}\right)\right)}}^{\frac{1}{Ⱥ}}}e^{2i\pi \left(\sqrt{1-{e^{-\left(\sum_{ɤ=1}^{ƞ}\left({Ϗ}_{ɤ}{\left(-\log\left(1-{\left(\emptyset_{ῠ}^{-}\right)}^{2}\right)\right)}^{Ⱥ}\right)\right)}}^{\frac{1}{Ⱥ}}}\right)}\leq$$

$$\sqrt{1-{e^{-\left(\sum_{ɤ=1}^{ƞ}\left({Ϗ}_{ɤ}{\left(-\log\left(1-\alpha_{ɤ}^{2}\right)\right)}^{Ⱥ}\right)\right)}}^{\frac{1}{Ⱥ}}}e^{2i\pi \left(\sqrt{1-{e^{-\left(\sum_{ɤ=1}^{ƞ}\left({Ϗ}_{ɤ}{\left(-\log\left(1-\emptyset_{ɤ}^{2}\right)\right)}^{Ⱥ}\right)\right)}}^{\frac{1}{Ⱥ}}}\right)}\leq$$

$$\sqrt{1-{e^{-\left(\sum_{ɤ=1}^{ƞ}\left({Ϗ}_{ɤ}{\left(-\log\left(1-{\left(\alpha_{ῠ}^{+}\right)}^{2}\right)\right)}^{Ⱥ}\right)\right)}}^{\frac{1}{Ⱥ}}}e^{2i\pi \left(\sqrt{1-{e^{-\left(\sum_{ɤ=1}^{ƞ}\left({Ϗ}_{ɤ}{\left(-\log\left(1-{\left(\emptyset_{ῠ}^{+}\right)}^{2}\right)\right)}^{Ⱥ}\right)\right)}}^{\frac{1}{Ⱥ}}}\right),}$$

and

$$e^{-{\left(\sum_{ɤ=1}^{ƞ}\left({{Ϗ}_{ɤ}\left(-\log\left(\nu_{ῠ}^{-}\right)\right)}^{Ⱥ}\right)\right)}^{\frac{1}{Ⱥ}}}e^{2i\pi \left({e^{-\left(\sum_{ɤ=1}^{ƞ}\left({{Ϗ}_{ɤ}\left(-\log\left(\varphi_{ῠ}^{-}\right)\right)}^{Ⱥ}\right)\right)}}^{\frac{1}{Ⱥ}}\right)}\geq e^{-{\left(\sum_{ɤ=1}^{ƞ}\left({{Ϗ}_{ɤ}\left(-\log\left(\nu_{ɤ}\right)\right)}^{Ⱥ}\right)\right)}^{\frac{1}{Ⱥ}}}e^{2i\pi \left({e^{-\left(\sum_{ɤ=1}^{ƞ}\left({{Ϗ}_{ɤ}\left(-\log\left(\varphi_{ɤ}\right)\right)}^{Ⱥ}\right)\right)}}^{\frac{1}{Ⱥ}}\right)}\geq e^{-{\left(\sum_{ɤ=1}^{ƞ}\left({{Ϗ}_{ɤ}\left(-\log\left(\nu_{ῠ}^{+}\right)\right)}^{Ⱥ}\right)\right)}^{\frac{1}{Ⱥ}}}e^{2i\pi \left({e^{-\left(\sum_{ɤ=1}^{ƞ}\left({{Ϗ}_{ɤ}\left(-\log\left(\varphi_{ῠ}^{+}\right)\right)}^{Ⱥ}\right)\right)}}^{\frac{1}{Ⱥ}}\right)}$$

$${e^{-\left(\sum_{ɤ=1}^{ƞ}{Ϗ}_{ɤ}{\left(-\log\left(\beta_{ῠ}^{-}\right)\right)}^{Ⱥ}\right)}}^{\frac{1}{Ⱥ}}e^{2i\pi \left({e^{-\left(\sum_{ɤ=1}^{ƞ}{{Ϗ}_{ɤ}\left(-\log\left(\left(\omega_{ῠ}^{-}\right)\right)\right)}^{Ⱥ}\right)}}^{\frac{1}{Ⱥ}}\right)}\geq {e^{-\left(\sum_{ɤ=1}^{ƞ}{Ϗ}_{ɤ}{\left(-\log\left(\beta_{ɤ}\right)\right)}^{Ⱥ}\right)}}^{\frac{1}{Ⱥ}}e^{2i\pi \left({e^{-\left(\sum_{ɤ=1}^{ƞ}{{Ϗ}_{ɤ}\left(-\log\left(\omega_{ɤ}\right)\right)}^{Ⱥ}\right)}}^{\frac{1}{Ⱥ}}\right)}\geq {e^{-\left(\sum_{ɤ=1}^{ƞ}{Ϗ}_{ɤ}{\left(-\log\left(\beta_{ῠ}^{+}\right)\right)}^{Ⱥ}\right)}}^{\frac{1}{Ⱥ}}e^{2i\pi \left({e^{-\left(\sum_{ɤ=1}^{ƞ}{{Ϗ}_{ɤ}\left(-\log\left(\left(\omega_{ῠ}^{+}\right)\right)\right)}^{Ⱥ}\right)}}^{\frac{1}{Ⱥ}}\right)}$$

*Therefore*:

$${ῠ}^{-}\leq CSFAAWA\left({ῠ}_{1},{ῠ}_{2},{ῠ}_{3},\ldots ,{ῠ}_{ƞ}\right)\leq {ῠ}^{+}$$

Theorem 4 *Let*${ῠ}_{ɤ}$*and*${ῠ}_{ɤ}^{\prime }$*are two sets of CSFVs*, *if*${ῠ}_{ɤ}\leq {ῠ}_{ɤ}^{\prime },\forall ,ɤ$. *Then we have*:

$$CSFAAWA\left({ῠ}_{1},{ῠ}_{2},{ῠ}_{3},\ldots ,{ῠ}_{ƞ}\right)\leq CSFAAWA\left({ῠ}_{1}^{\prime },{ῠ}_{2}^{\prime },{ῠ}_{3}^{\prime },\ldots ,{ῠ}_{ƞ}^{\prime }\right)$$

Definition 8 Consider ${ῠ}_{ɤ}=\left(\alpha_{ɤ}e^{2i\pi \left(\emptyset_{ɤ}\right)},\nu_{ɤ}e^{2i\pi \left(\varphi_{ɤ}\right)},\beta_{ɤ}e^{2i\pi \left(\omega_{ɤ}\right)}\right),ɤ=1,2,3,\ldots ,ƞ$ be the set of CSFVs. Then, the CSFAAOWA operator is given as:

$$CSFAAOWA\left({ῠ}_{1},{ῠ}_{2},\ldots ,{ῠ}_{ƞ}\right)={⊕}_{ɤ=1}^{ƞ}\left({Ϗ}_{ɤ}{ῠ}_{Ϸ\left(ɤ\right)}\right)={Ϗ}_{1}{ῠ}_{Ϸ\left(1\right)}⨁{Ϗ}_{2}{ῠ}_{Ϸ\left(2\right)}⨁,\ldots ,⨁{Ϗ}_{ƞ}{ῠ}_{Ϸ\left(ƞ\right)}$$

where (Ϸ(1),Ϸ(2),Ϸ(3),…,Ϸ(ɤ)) be a permutation of (ɤ=1,2,3,…ƞ) and ${ῠ}_{Ϸ\left(ɤ-1\right)}\geq {ῠ}_{Ϸ\left(ɤ\right)},\forall ,ɤ=1,2,3,\ldots ƞ$.

Theorem 5 Let ${ῠ}_{ɤ}=\left(\alpha_{ɤ}e^{2i\pi \left(\emptyset_{ɤ}\right)},\nu_{ɤ}e^{2i\pi \left(\varphi_{ɤ}\right)},\beta_{ɤ}e^{2i\pi \left(\omega_{ɤ}\right)}\right),ɤ=1,2,3,\ldots ,ƞ$ be the set of CSFVs. Then, the associated values of the CSFAAOWA operator are particularized as:

$$CSFAAOWA\left({ῠ}_{1},{ῠ}_{2},\ldots ,{ῠ}_{ƞ}\right)=\left(\begin{array}{c} \sqrt{1-e^{-{\left({\sum_{ɤ=1}^{ƞ}{Ϗ}_{ɤ}\left(-\log\left(1-\alpha_{Ϸ\left(ɤ\right)}^{2}\right)\right)}^{Ⱥ}\right)}^{\frac{1}{Ⱥ}}}e^{2\pi i\left(\sqrt{1-e^{-{\left({\sum_{ɤ=1}^{ƞ}{Ϗ}_{ɤ}\left(-\log\left(1-\emptyset_{Ϸ\left(ɤ\right)}^{2}\right)\right)}^{Ⱥ}\right)}^{\frac{1}{Ⱥ}}}}\right)}},\\ e^{-{\left(\sum_{ɤ=1}^{ƞ}{Ϗ}_{ɤ}{\left(-\log\left(\nu_{Ϸ\left(ɤ\right)}\right)\right)}^{Ⱥ}\right)}^{\frac{1}{Ⱥ}}}e^{2\pi i\left(e^{-{\left(\sum_{ɤ=1}^{ƞ}{Ϗ}_{ɤ}{\left(-\log\left(\varphi_{Ϸ\left(ɤ\right)}\right)\right)}^{Ⱥ}\right)}^{\frac{1}{Ⱥ}}}\right)},\\ e^{-{\left(\sum_{ɤ=1}^{ƞ}{Ϗ}_{ɤ}{\left(-\log\left(\beta_{Ϸ\left(ɤ\right)}\right)\right)}^{Ⱥ}\right)}^{\frac{1}{Ⱥ}}e^{2\pi i\left(e^{-{\left(\sum_{ɤ=1}^{ƞ}{Ϗ}_{ɤ}{\left(-\log\left(\omega_{Ϸ\left(ɤ\right)}\right)\right)}^{Ⱥ}\right)}^{\frac{1}{Ⱥ}}}\right)}} \end{array}\right)$$

where (Ϸ(1),Ϸ(2),Ϸ(3),…,Ϸ(ɤ)) be the set of permutations of (ɤ=1,2,3,…,ƞ) and ${ῠ}_{Ϸ\left(ɤ-1\right)}\geq {ῠ}_{Ϸ\left(ɤ\right)},\forall ,ɤ=1,2,3,\ldots ƞ$.

Theorem 6 *Consider*${ῠ}_{ɤ}=\left(\alpha_{ɤ}e^{2i\pi \left(\emptyset_{ɤ}\right)},\nu_{ɤ}e^{2i\pi \left(\varphi_{ɤ}\right)},\beta_{ɤ}e^{2i\pi \left(\omega_{ɤ}\right)}\right),ɤ=1,2,3,\ldots ,ƞ\text{,}$*be the family of all same CSFVs*, ∀,ɤ=1,2,…,ƞ.*Then we have*:

$$CSFAAOWA\left({ῠ}_{1},{ῠ}_{2},\ldots ,{ῠ}_{ƞ}\right)=ῠ$$

Theorem 7 *Let*${ῠ}_{ɤ}=\left(\alpha_{ɤ}e^{2i\pi \left(\emptyset_{ɤ}\right)},\nu_{ɤ}e^{2i\pi \left(\varphi_{ɤ}\right)},\beta_{ɤ}e^{2i\pi \left(\omega_{ɤ}\right)}\right),ɤ=1,2,3,\ldots ,ƞ$*as the family of CSFVs*, *and*${ῠ}^{-}=\min\left({ῠ}_{1},{ῠ}_{2},{ῠ}_{3},\ldots ,{ῠ}_{ƞ}\right)$*and*${ῠ}^{+}=\max\left({ῠ}_{1},{ῠ}_{2},{ῠ}_{3},\ldots ,{ῠ}_{ƞ}\right)$. *Then*, *we can get*:

$${ῠ}^{-}\leq CSFAAOWA\left({ῠ}_{1},{ῠ}_{2},\ldots ,{ῠ}_{ƞ}\right)\leq {ῠ}^{+}$$

Theorem 8 *Consider*${ῠ}_{ɤ}=\left(\alpha_{ɤ}e^{2i\pi \left(\emptyset_{ɤ}\right)},\nu_{ɤ}e^{2i\pi \left(\varphi_{ɤ}\right)},\beta_{ɤ}e^{2i\pi \left(\omega_{ɤ}\right)}\right),ɤ=1,2,3,\ldots ,ƞ\text{,}$*and*${ῠ}_{ɤ}=\left(\alpha_{ɤ}e^{2i\pi \left(\emptyset_{ɤ}\right)},\nu_{ɤ}e^{2i\pi \left(\varphi_{ɤ}\right)},\beta_{ɤ}e^{2i\pi \left(\omega_{ɤ}\right)}\right),ɤ=1,2,3,\ldots ,ƞ$*are two CSFSs and if*${ῠ}_{ɤ}\leq {ῠ}_{ɤ}^{\prime },\forall ,\left(ɤ=1,2,\ldots ,ƞ\right)$. *Then*, *we have*:

$$CSFAAOWA\left({ῠ}_{1},{ῠ}_{2},\ldots ,{ῠ}_{ƞ}\right)\leq CSFAAOWA\left({ῠ}_{1}^{\prime },{ῠ}_{2}^{\prime },\ldots ,{ῠ}_{ƞ}^{\prime }\right)$$

## 5 Complex spherical fuzzy Aczel-Alsina aggregation operators

In this section, some geometric aggregation tools of CSFVs were developed by using the basic operations of Aczel-Alsina aggregation tools.

Definition 9 Consider ${ῠ}_{ɤ}=\left(\alpha_{ɤ}e^{2i\pi \left(\emptyset_{ɤ}\right)},\nu_{ɤ}e^{2i\pi \left(\varphi_{ɤ}\right)},\beta_{ɤ}e^{2i\pi \left(\omega_{ɤ}\right)}\right),ɤ=1,2,3,\ldots ,ƞ$ be the set of CSFVs. Then, the CSFAAWG operator is given as:

$$CSFAAWG\left({ῠ}_{1},{ῠ}_{2},\ldots ,{ῠ}_{ƞ}\right)=\overset{ƞ}{\underset{ɤ=1}{⨂}}\left({ῠ}_{ɤ}^{{Ϗ}_{ɤ}}\right)={ῠ}_{1}^{{Ϗ}_{1}}⨂{ῠ}_{2}^{{Ϗ}_{2}}⨂,\ldots ,⨂{ῠ}_{ƞ}^{{Ϗ}_{ƞ}}$$

Theorem 9 *Consider*${ῠ}_{ɤ}=\left(\alpha_{ɤ}e^{2i\pi \left(\emptyset_{ɤ}\right)},\nu_{ɤ}e^{2i\pi \left(\varphi_{ɤ}\right)},\beta_{ɤ}e^{2i\pi \left(\omega_{ɤ}\right)}\right),ɤ=1,2,3,\ldots ,ƞ$*be the set of CSFVs*. *Then*, *the integrated values of the CSFAAWG operator are also a CSFV*, *we have*:

$$CSFAAWG\left({ῠ}_{1},{ῠ}_{2},\ldots ,{ῠ}_{ƞ}\right)=\left(\begin{array}{c} {e^{-\left(\sum_{ɤ=1}^{ƞ}{Ϗ}_{ɤ}{\left(-\log\left(\alpha_{ɤ}\right)\right)}^{Ⱥ}\right)}}^{\frac{1}{Ⱥ}}e^{2i\pi \left({e^{-\left(\sum_{ɤ=1}^{ƞ}{{Ϗ}_{ɤ}\left(-\log\left(\emptyset_{ɤ}\right)\right)}^{Ⱥ}\right)}}^{\frac{1}{Ⱥ}}\right)},\\ \sqrt{1-{e^{-\left(\sum_{ɤ=1}^{ƞ}\left({Ϗ}_{ɤ}{\left(-\log\left(1-\nu_{ɤ}^{2}\right)\right)}^{Ⱥ}\right)\right)}}^{\frac{1}{Ⱥ}}}e^{2i\pi \left(\sqrt{1-{e^{-\left(\sum_{ɤ=1}^{ƞ}\left({Ϗ}_{ɤ}{\left(-\log\left(1-\varphi_{ɤ}^{2}\right)\right)}^{Ⱥ}\right)\right)}}^{\frac{1}{Ⱥ}}}\right)}\\ \sqrt{1-{e^{-\left(\sum_{ɤ=1}^{ƞ}\left({Ϗ}_{ɤ}{\left(-\log\left(1-\beta_{ɤ}^{2}\right)\right)}^{Ⱥ}\right)\right)}}^{\frac{1}{Ⱥ}}}e^{2i\pi \left(\sqrt{1-{e^{-\left(\sum_{ɤ=1}^{ƞ}\left({Ϗ}_{ɤ}{\left(-\log\left(1-\omega_{ɤ}^{2}\right)\right)}^{Ⱥ}\right)\right)}}^{\frac{1}{Ⱥ}}}\right)} \end{array}\right)$$

Theorem 10 *Consider*${ῠ}_{ɤ}=\left(\alpha_{ɤ}e^{2i\pi \left(\emptyset_{ɤ}\right)},\nu_{ɤ}e^{2i\pi \left(\varphi_{ɤ}\right)},\beta_{ɤ}e^{2i\pi \left(\omega_{ɤ}\right)}\right),ɤ=1,2,3,\ldots ,ƞ\text{,}$*be the set of all same CSFVs*, ∀,ɤ=1,2,…,ƞ.*Then*, *we have*:

$$CSFAAWG\left({ῠ}_{1},{ῠ}_{2},\ldots ,{ῠ}_{ƞ}\right)=ῠ$$

Theorem 11 *Let*${ῠ}_{ɤ}=\left(\alpha_{ɤ}e^{2i\pi \left(\emptyset_{ɤ}\right)},\nu_{ɤ}e^{2i\pi \left(\varphi_{ɤ}\right)},\beta_{ɤ}e^{2i\pi \left(\omega_{ɤ}\right)}\right),ɤ=1,2,3,\ldots ,ƞ$*be the family of CSFVs*, *and*${ῠ}^{-}=\min\left({ῠ}_{1},{ῠ}_{2},{ῠ}_{3},\ldots ,{ῠ}_{ƞ}\right)$*and*${ῠ}^{+}=\max\left({ῠ}_{1},{ῠ}_{2},{ῠ}_{3},\ldots ,{ῠ}_{ƞ}\right)$. *Then*, *the associated value*$CSFAAWG\left({ῠ}_{1},{ῠ}_{2},\ldots ,{ῠ}_{k}\right)$*has that*

$${ῠ}^{-}\leq CSFAAWG\left({ῠ}_{1},{ῠ}_{2},\ldots ,{ῠ}_{ƞ}\right)\leq {ῠ}^{+}$$

Theorem 12 *Let*${ῠ}_{ɤ}=\left(\alpha_{ɤ}e^{2i\pi \left(\emptyset_{ɤ}\right)},\nu_{ɤ}e^{2i\pi \left(\varphi_{ɤ}\right)},\beta_{ɤ}e^{2i\pi \left(\omega_{ɤ}\right)}\right),ɤ=1,2,3,\ldots ,ƞ$*and*${ῠ}_{ɤ}=\left(\alpha_{ɤ}e^{2i\pi \left(\emptyset_{ɤ}\right)},\nu_{ɤ}e^{2i\pi \left(\varphi_{ɤ}\right)},\beta_{ɤ}e^{2i\pi \left(\omega_{ɤ}\right)}\right),ɤ=1,2,3,\ldots ,ƞ$*are two CSFSs and if*${ῠ}_{ɤ}\leq {ῠ}_{ɤ}^{\prime },\forall ,\left(ɤ=1,2,\ldots ,ƞ\right)\text{.}$*Then we have*:

$$CSFAAWG\left({ῠ}_{1},{ῠ}_{2},\ldots ,{ῠ}_{ƞ}\right)\leq CSFAAWG\left({ῠ}_{1}^{\prime },{ῠ}_{2}^{\prime },\ldots ,{ῠ}_{ƞ}^{\prime }\right)$$

Example 2 Consider ${ῠ}_{1}=\left(0.51e^{2i\pi \left(0.32\right)},0.39e^{2i\pi \left(0.71\right)},0.45e^{2i\pi \left(0.39\right)}\right),{ῠ}_{2}=\left(0.27e^{2i\pi \left(0.33\right)},0.45e^{2i\pi \left(0.61\right)},0.09e^{2i\pi \left(0.37\right)}\right),{ῠ}_{3}=\left(0.55e^{2i\pi \left(0.78\right)},0.18e^{2i\pi \left(0.28\right)},0.45e^{2i\pi \left(0.51\right)}\right)$ and ${ῠ}_{4}=\left(0.29e^{2i\pi \left(0.57\right)},0.16e^{2i\pi \left(0.48\right)},0.87e^{2i\pi \left(0.55\right)}\right)$ are four CSFVs and let Ϗ=(0.23,0.27,0.30,0.20) be the weight vector of the CSFVs. Then the solution of given CSFVs obtained by the CSFAAWG operators is given as suppose Ⱥ=3.

Solution:

$$CSFAAWG\left({ῠ}_{1},{ῠ}_{2},\ldots ,{ῠ}_{ƞ}\right)=\left(\begin{array}{c} {e^{-\left(\sum_{ɤ=1}^{ƞ}{Ϗ}_{ɤ}{\left(-\log\left(\alpha_{ɤ}\right)\right)}^{Ⱥ}\right)}}^{\frac{1}{Ⱥ}}e^{2i\pi \left({e^{-\left(\sum_{ɤ=1}^{ƞ}{{Ϗ}_{ɤ}\left(-\log\left(\emptyset_{ɤ}\right)\right)}^{Ⱥ}\right)}}^{\frac{1}{Ⱥ}}\right)},\\ \sqrt{1-{e^{-\left(\sum_{ɤ=1}^{ƞ}\left({Ϗ}_{ɤ}{\left(-\log\left(1-\nu_{ɤ}^{2}\right)\right)}^{Ⱥ}\right)\right)}}^{\frac{1}{Ⱥ}}}e^{2i\pi \left(\sqrt{1-{e^{-\left(\sum_{ɤ=1}^{ƞ}\left({Ϗ}_{ɤ}{\left(-\log\left(1-\varphi_{ɤ}^{2}\right)\right)}^{Ⱥ}\right)\right)}}^{\frac{1}{Ⱥ}}}\right)}\\ \sqrt{1-{e^{-\left(\sum_{ɤ=1}^{ƞ}\left({Ϗ}_{ɤ}{\left(-\log\left(1-\beta_{ɤ}^{2}\right)\right)}^{Ⱥ}\right)\right)}}^{\frac{1}{Ⱥ}}}e^{2i\pi \left(\sqrt{1-{e^{-\left(\sum_{ɤ=1}^{ƞ}\left({Ϗ}_{ɤ}{\left(-\log\left(1-\omega_{ɤ}^{2}\right)\right)}^{Ⱥ}\right)\right)}}^{\frac{1}{Ⱥ}}}\right)} \end{array}\right)$$

$$CSFAAWG\left({ῠ}_{1},{ῠ}_{2},{ῠ}_{3},{ῠ}_{4}\right)=\left(\begin{array}{c} e^{-{\left(\left(\begin{array}{c} {\left(0.23\right)\left(-\log\left(0.51\right)\right)}^{3}+{\left(0.27\right)\left(-\log\left(0.27\right)\right)}^{3}+\\ {\left(0.30\right)\left(-\log\left(0.55\right)\right)}^{3}+{\left(0.20\right)\left(-\log\left(0.29\right)\right)}^{3} \end{array}\right)\right)}^{\frac{1}{3}}}\\ e^{2\pi i\left(e^{-{\left(\left(\begin{array}{c} {\left(0.23\right)\left(-\log\left(0.32\right)\right)}^{3}+{\left(0.27\right)\left(-\log\left(0.33\right)\right)}^{3}+\\ {\left(0.30\right)\left(-\log\left(0.78\right)\right)}^{3}+{\left(0.20\right)\left(-\log\left(0.57\right)\right)}^{3} \end{array}\right)\right)}^{\frac{1}{3}}}\right)},\\ \sqrt{1-e^{-{\left(\left(\begin{array}{c} \left(0.23\right){\left(-\log\left(1-{\left(0.39\right)}^{2}\right)\right)}^{3}+\left(0.27\right){\left(-\log\left(1-{\left(0.45\right)}^{2}\right)\right)}^{3}+\\ \left(0.30\right){\left(-\log\left(1-{\left(0.18\right)}^{2}\right)\right)}^{3}+\left(0.20\right){\left(-\log\left(1-{\left(0.16\right)}^{2}\right)\right)}^{3} \end{array}\right)\right)}^{\frac{1}{3}}}}\\ e^{2\pi i\left(\sqrt{1-e^{-{\left(\left(\begin{array}{c} \left(0.23\right){\left(-\log\left(1-{\left(0.71\right)}^{2}\right)\right)}^{3}+\left(0.27\right){\left(-\log\left(1-{\left(0.61\right)}^{2}\right)\right)}^{3}+\\ \left(0.30\right){\left(-\log\left(1-{\left(0.28\right)}^{2}\right)\right)}^{3}+\left(0.20\right){\left(-\log\left(1-{\left(0.48\right)}^{2}\right)\right)}^{3} \end{array}\right)\right)}^{\frac{1}{3}}}}\right)},\\ \sqrt{1-e^{-{\left(\left(\begin{array}{c} \left(0.23\right){\left(-\log\left(1-{\left(0.45\right)}^{2}\right)\right)}^{3}+\left(0.27\right){\left(-\log\left(1-{\left(0.09\right)}^{2}\right)\right)}^{3}+\\ \left(0.30\right){\left(-\log\left(1-{\left(0.45\right)}^{2}\right)\right)}^{3}+\left(0.20\right){\left(-\log\left(1-{\left(0.87\right)}^{2}\right)\right)}^{3} \end{array}\right)\right)}^{\frac{1}{3}}}}\\ e^{2\pi i\left(\sqrt{1-e^{-{\left(\left(\begin{array}{c} \left(0.23\right){\left(-\log\left(1-{\left(0.39\right)}^{2}\right)\right)}^{3}+\left(0.27\right){\left(-\log\left(1-{\left(0.37\right)}^{2}\right)\right)}^{3}+\\ \left(0.30\right){\left(-\log\left(1-{\left(0.51\right)}^{2}\right)\right)}^{3}+\left(0.20\right){\left(-\log\left(1-{\left(0.55\right)}^{2}\right)\right)}^{3} \end{array}\right)\right)}^{\frac{1}{3}}}}\right)} \end{array}\right)$$

$$=\left(0.6370e^{2i\pi \left(0.6742\right)},0.2599e^{2i\pi \left(0.4336\right)},0.5502e^{2i\pi \left(0.3320\right)}\right)$$

Definition 10 *Let*${ῠ}_{ɤ}=\left(\alpha_{ɤ}e^{2i\pi \left(\emptyset_{ɤ}\right)},\nu_{ɤ}e^{2i\pi \left(\varphi_{ɤ}\right)},\beta_{ɤ}e^{2i\pi \left(\omega_{ɤ}\right)}\right),ɤ=1,2,3,\ldots ,ƞ$*be the set of CSFVs*. *Then*, *associated values of the CSFAAOWG operator are given as*:

$$CSFAAOWG\left({ῠ}_{1},{ῠ}_{2},\ldots ,{ῠ}_{ƞ}\right)=\overset{ƞ}{\underset{ɤ=1}{⨂}}\left({ῠ}_{ƺ\left(ɤ\right)}^{{Ϗ}_{ɤ}}\right)={ῠ}_{ƺ\left(1\right)}^{{Ϗ}_{1}}⨂{ῠ}_{ƺ\left(2\right)}^{{Ϗ}_{2}}⨂,\ldots ,⨂{ῠ}_{ƺ\left(ƞ\right)}^{{Ϗ}_{ƞ}}$$

where the set of permutations is denoted by (ƺ(1),ƺ(2),ƺ(3),…,ƺ(ɤ)) of (ɤ=1,2,3,…ƞ) and ${ῠ}_{ƺ\left(ɤ-1\right)}\geq {ῠ}_{ƺ\left(ɤ\right)},\forall ,ɤ=1,2,3,\ldots ƞ$.

Theorem 13 Let ${ῠ}_{ɤ}=\left(\alpha_{ɤ}e^{2i\pi \left(\emptyset_{ɤ}\right)},\nu_{ɤ}e^{2i\pi \left(\varphi_{ɤ}\right)},\beta_{ɤ}e^{2i\pi \left(\omega_{ɤ}\right)}\right),ɤ=1,2,3,\ldots ,ƞ$ be the set of CSFVs. Then the integrated values of the CSFAAOWG operator are also a CSFV, we have:

$$CSFAAOWG\left({ῠ}_{1},{ῠ}_{2},\ldots ,{ῠ}_{ƞ}\right)=\left(\begin{array}{c} e^{-{\left(\sum_{ɤ=1}^{ƞ}{Ϗ}_{ɤ}{\left(-\log\left(\alpha_{Ϸ\left(ɤ\right)}\right)\right)}^{Ⱥ}\right)}^{\frac{1}{Ⱥ}}}e^{2\pi i\left(e^{-{\left(\sum_{ɤ=1}^{ƞ}{Ϗ}_{ɤ}{\left(-\log\left(\emptyset_{Ϸ\left(ɤ\right)}\right)\right)}^{Ⱥ}\right)}^{\frac{1}{Ⱥ}}}\right)},\\ \sqrt{1-e^{-{\left({\sum_{ɤ=1}^{ƞ}{Ϗ}_{ɤ}\left(-\log\left(1-\nu_{Ϸ\left(ɤ\right)}^{2}\right)\right)}^{Ⱥ}\right)}^{\frac{1}{Ⱥ}}}e^{2\pi i\left(\sqrt{1-e^{-{\left({\sum_{ɤ=1}^{ƞ}{Ϗ}_{ɤ}\left(-\log\left(1-\varphi_{Ϸ\left(ɤ\right)}^{2}\right)\right)}^{Ⱥ}\right)}^{\frac{1}{Ⱥ}}}}\right)}},\\ \sqrt{1-e^{-{\left({\sum_{ɤ=1}^{ƞ}{Ϗ}_{ɤ}\left(-\log\left(1-\beta_{Ϸ\left(ɤ\right)}^{2}\right)\right)}^{Ⱥ}\right)}^{\frac{1}{Ⱥ}}}e^{2\pi i\left(\sqrt{1-e^{-{\left({\sum_{ɤ=1}^{ƞ}{Ϗ}_{ɤ}\left(-\log\left(1-\omega_{Ϸ\left(ɤ\right)}^{2}\right)\right)}^{Ⱥ}\right)}^{\frac{1}{Ⱥ}}}}\right)}} \end{array}\right)$$

where (Ϸ(1),Ϸ(2),Ϸ(3),…,Ϸ(ɤ)) be the set of permutations of ${ῠ}_{ɤ},\left(ɤ=1,2,3,\ldots ,ƞ\right)$.

Theorem 14 Consider ${ῠ}_{ɤ}=\left(\alpha_{ɤ}e^{2i\pi \left(\emptyset_{ɤ}\right)},\nu_{ɤ}e^{2i\pi \left(\varphi_{ɤ}\right)},\beta_{ɤ}e^{2i\pi \left(\omega_{ɤ}\right)}\right),ɤ=1,2,3,\ldots ,ƞ\text{,}$ be the family of all same CSFVs, ∀,ɤ=1,2,…,ƞ. Then we have:

$$CSFAAOWA\left({ῠ}_{1},{ῠ}_{2},\ldots ,{ῠ}_{ƞ}\right)=ῠ$$

Theorem 15 *Let*${ῠ}_{ɤ}=\left(\alpha_{ɤ}e^{2i\pi \left(\emptyset_{ɤ}\right)},\nu_{ɤ}e^{2i\pi \left(\varphi_{ɤ}\right)},\beta_{ɤ}e^{2i\pi \left(\omega_{ɤ}\right)}\right),ɤ=1,2,3,\ldots ,ƞ$*as the family of CSFVs*, *and*${ῠ}^{-}=\min\left({ῠ}_{1},{ῠ}_{2},{ῠ}_{3},\ldots ,{ῠ}_{ƞ}\right)$*and*${ῠ}^{+}=\max\left({ῠ}_{1},{ῠ}_{2},{ῠ}_{3},\ldots ,{ῠ}_{ƞ}\right)$. *Then*, *we can get*:

$${ῠ}^{-}\leq CSFAAOWA\left({ῠ}_{1},{ῠ}_{2},\ldots ,{ῠ}_{ƞ}\right)\leq {ῠ}^{+}$$

Theorem 16 *Let*${ῠ}_{ɤ}=\left(\alpha_{ɤ}e^{2i\pi \left(\emptyset_{ɤ}\right)},\nu_{ɤ}e^{2i\pi \left(\varphi_{ɤ}\right)},\beta_{ɤ}e^{2i\pi \left(\omega_{ɤ}\right)}\right),ɤ=1,2,3,\ldots ,ƞ\text{,}$*and*${ῠ}_{ɤ}^{\prime }=\left(\alpha_{ɤ}^{\prime }e^{2i\pi \left(\emptyset_{ɤ}^{\prime }\right)},\nu_{ɤ}e^{2i\pi \left(\varphi_{ɤ}\right)},\beta_{ɤ}e^{2i\pi \left(\omega_{ɤ}\right)}\right),ɤ=1,2,3,\ldots ,ƞ$*are two CSFSs and if*${ῠ}_{ɤ}\leq {ῠ}_{ɤ}^{\prime },\forall ,\left(ɤ=1,2,\ldots ,ƞ\right)$. *Then*, *we have*:

$$CSFAAOWA\left({ῠ}_{1},{ῠ}_{2},\ldots ,{ῠ}_{ƞ}\right)\leq CSFAAOWA\left({ῠ}_{1}^{\prime },{ῠ}_{2}^{\prime },\ldots ,{ῠ}_{ƞ}^{\prime }\right)$$

where (Ϸ(1),Ϸ(2),Ϸ(3),…,Ϸ(ɤ)) be the set of permutations of $\left({ῠ}_{ɤ}^{\prime }:ɤ=1,2,3,\ldots ,ƞ\right)$.

## 6 Assessment of the MCDM technique based on CSF information

Now we evaluate the MCDM problem by utilizing our presented methodologies such as CSFAAWA and CSFAAWG operators. Our developed methodologies are used to integrate the collection of information, after a lot of investigation and computation of the information we get a single value for a strong decision purpose. These discussed approaches assess information more conveniently and accurately.

Consider a set ẞ of alternatives, which contains ῥ different types of alternatives like $\left\{A_{1},A_{2},\ldots ,A_{ῥ}\right\}$ and a set of attributes Ế contains ҙ distinguish characteristics like $\left\{J_{1},J_{2},\ldots ,J_{ҙ}\right\}$. A decision maker evaluates information based on certain degrees assigned to each characteristic by the experts. Assume a set of characteristics degree indicated by the ${đ}_{s}=\left({đ}_{1},{đ}_{2},\ldots ,{đ}_{ҙ}\right)$ such that ${đ}_{s}>0$ and $\sum_{s=1}^{ҙ}{đ}_{i}=1$. Decision makers assess the information of CSFVs ${ῠ}_{ps}=\left(\alpha_{ps}e^{2i\pi \left(\emptyset_{ps}\right)},\nu_{ps}e^{2i\pi \left(\varphi_{ps}\right)},\beta_{ps}e^{2i\pi \left(\omega_{ps}\right)}\right),p=1,2,\ldots ,ῥ$ and s=1,2,…,ҙ based on assigning degrees of characteristics by the experts. Each CSFV satisfy such condition $0\leq \alpha_{ps}+\nu_{ps}+\beta_{ps}\leq 1$ and $0\leq \emptyset_{ps}+\varphi_{ps}+\omega_{ps}\leq 1$. The decision maker constructed CSF information in the decision matrix based on some essential components of information like alternatives and attributes. By utilizing proposed methodologies with assigning degrees of characteristics, experts evaluated the collection of information and compute the results of individuals based on different features in a single term. In this article, we have used hypothetical information about the discussed application of an electric motor car. We explored an algorithm to evaluate the decision-making problem by using the following steps of an algorithm under our current presented approaches.

Step 1 Each alternative contains information in the form of CSFVs given by the decision maker, to assess this information under some criteria. All collected information is drawn in the following decision matrix:

$$E={\left({ῠ}_{ps}\right)}_{ῥ\times ҙ}=\left[\begin{array}{cc} \begin{array}{ccc} \begin{array}{c} {ῠ}_{11}\\ {ῠ}_{21}\\ \begin{array}{c} \vdots \\ {ῠ}_{ῥ1} \end{array} \end{array} & \begin{array}{c} {ῠ}_{12}\\ {ῠ}_{22}\\ \begin{array}{c} \vdots \\ {ῠ}_{ῥ2} \end{array} \end{array} & \begin{array}{c} \cdots \\ \cdots \\ \begin{array}{c} \ddots \\ \cdots \end{array} \end{array} \end{array} & \begin{array}{c} {ῠ}_{1ҙ}\\ {ῠ}_{2ҙ}\\ \begin{array}{c} \vdots \\ {ῠ}_{ῥҙ} \end{array} \end{array} \end{array}\right]$$

where ${\left({ῠ}_{ps}\right)}_{ῥ\times ҙ}={\left(\alpha_{ps}e^{2i\pi \left(\emptyset_{ps}\right)},\nu_{ps}e^{2i\pi \left(\varphi_{ps}\right)},\beta_{ps}e^{2i\pi \left(\omega_{ps}\right)}\right)}_{ῥ\times ҙ},p=1,2,\ldots ,ῥ$ and s=1,2,…,ҙ.

Step 2 integrate each attribute corresponding to each alternative based on certain criteria by utilizing invented approaches of the CSFAAWA and CSFAAWG operators.

$$CSFAAWA\left({ῠ}_{p1},{ῠ}_{p2},\ldots ,{ῠ}_{ps}\right)=\left(\begin{array}{c} \sqrt{1-{e^{-\left(\sum_{ɤ=1}^{ƞ}\left({Ϗ}_{ɤ}{\left(-\log\left(1-\alpha_{ɤ}^{2}\right)\right)}^{Ⱥ}\right)\right)}}^{\frac{1}{Ⱥ}}}e^{2i\pi \left(\sqrt{1-{e^{-\left(\sum_{ɤ=1}^{ƞ}\left({Ϗ}_{ɤ}{\left(-\log\left(1-\emptyset_{ɤ}^{2}\right)\right)}^{Ⱥ}\right)\right)}}^{\frac{1}{Ⱥ}}}\right)},\\ e^{-{\left(\sum_{ɤ=1}^{ƞ}\left({{Ϗ}_{ɤ}\left(-\log\left(\nu_{ɤ}\right)\right)}^{Ⱥ}\right)\right)}^{\frac{1}{Ⱥ}}}e^{2i\pi \left({e^{-\left(\sum_{ɤ=1}^{ƞ}\left({{Ϗ}_{ɤ}\left(-\log\left(\varphi_{ɤ}\right)\right)}^{Ⱥ}\right)\right)}}^{\frac{1}{Ⱥ}}\right)},\\ {e^{-\left(\sum_{ɤ=1}^{ƞ}{Ϗ}_{ɤ}{\left(-\log\left(\beta_{ɤ}\right)\right)}^{Ⱥ}\right)}}^{\frac{1}{Ⱥ}}e^{2i\pi \left({e^{-\left(\sum_{ɤ=1}^{ƞ}{{Ϗ}_{ɤ}\left(-\log\left(\omega_{ɤ}\right)\right)}^{Ⱥ}\right)}}^{\frac{1}{Ⱥ}}\right)} \end{array}\right)$$

$$CSFAAWG\left({ῠ}_{p1},{ῠ}_{p2},\ldots ,{ῠ}_{ps}\right)=\left(\begin{array}{c} {e^{-\left(\sum_{ɤ=1}^{ƞ}{Ϗ}_{ɤ}{\left(-\log\left(\alpha_{ɤ}\right)\right)}^{Ⱥ}\right)}}^{\frac{1}{Ⱥ}}e^{2i\pi \left({e^{-\left(\sum_{ɤ=1}^{ƞ}{{Ϗ}_{ɤ}\left(-\log\left(\emptyset_{ɤ}\right)\right)}^{Ⱥ}\right)}}^{\frac{1}{Ⱥ}}\right)},\\ \sqrt{1-{e^{-\left(\sum_{ɤ=1}^{ƞ}\left({Ϗ}_{ɤ}{\left(-\log\left(1-\nu_{ɤ}^{2}\right)\right)}^{Ⱥ}\right)\right)}}^{\frac{1}{Ⱥ}}}e^{2i\pi \left(\sqrt{1-{e^{-\left(\sum_{ɤ=1}^{ƞ}\left({Ϗ}_{ɤ}{\left(-\log\left(1-\varphi_{ɤ}^{2}\right)\right)}^{Ⱥ}\right)\right)}}^{\frac{1}{Ⱥ}}}\right)}\\ \sqrt{1-{e^{-\left(\sum_{ɤ=1}^{ƞ}\left({Ϗ}_{ɤ}{\left(-\log\left(1-\beta_{ɤ}^{2}\right)\right)}^{Ⱥ}\right)\right)}}^{\frac{1}{Ⱥ}}}e^{2i\pi \left(\sqrt{1-{e^{-\left(\sum_{ɤ=1}^{ƞ}\left({Ϗ}_{ɤ}{\left(-\log\left(1-\omega_{ɤ}^{2}\right)\right)}^{Ⱥ}\right)\right)}}^{\frac{1}{Ⱥ}}}\right)} \end{array}\right)$$

Step 3 compute score values by using Definition 3 based on the consequences of the CSFAAWA and CSFAAWG operators.

Step 4 To evaluate a more appropriate alternative, rearrange all score values in descending order.

Step 5 End

### 6.1 Numerical example

Transportation is essential to modern life, but combustion engines are ageing. Fully electric vehicles quickly replace gasoline and diesel-powered vehicles since they are so much more environmentally friendly. Electric vehicles have significantly lower operating expenses than comparable gasoline or diesel vehicles. Electric vehicles use energy to charge their batteries as opposed to using fossil fuels like gasoline or diesel. Since charging an electric car is less expensive than purchasing gasoline or diesel to power it for our transportation needs, electric vehicles are more cost-effective than those. When renewable energy sources power electric vehicles, their use can be more environmentally friendly. The price of electricity can be further reduced if charging is done with the help of renewable energy sources installed at home, such as solar panels.

Consider five different types of electric cars $Ǽ=\left({Ǽ}_{1},{Ǽ}_{2},{Ǽ}_{3},{Ǽ}_{4},{Ǽ}_{5}\right)$ are available. The decision-maker assessment of these electric cars is based on four different types of characteristics such as: $J_{1}$ indicates the storage capacity of the batteries, $J_{2}$ represents the low maintenance expenditures, $J_{3}$ is a reliable and easy to operative system and $J_{4}$ less noise pollution rate and long-life warranty. All discussed characteristics are evaluated by using assigning degree (0.23,0.27,0.30,0.20) of the characteristics of the decision maker. The following decision matrix contains information about electric vehicles in the form of SFNs.

### 6.2 Assessment of decision-making problem

Step 1: Utilized the CSFAAWA and CSFAAWG operators and aggregated CSF information about electric motor cars drawn in (Table 2). Outcomes by the CSFAAWA and CSFAAWG operators are shown in (Table 3) and (Table 4) respectively.

**Table 2:** Contains information based on SFNs.

	$J_{1}$	$J_{2}$
${Ǽ}_{1}$	$\left(0.35e^{2i\pi \left(0.23\right)},0.46e^{2i\pi \left(0.71\right)},0.15e^{2i\pi \left(0.55\right)}\right)$	$\left(0.46e^{2i\pi \left(0.55\right)},0.45e^{2i\pi \left(0.09\right)},0.51e^{2i\pi \left(0.66\right)}\right)$
${Ǽ}_{2}$	$\left(0.46e^{2i\pi \left(0.33\right)},0.15e^{2i\pi \left(0.24\right)},0.61e^{2i\pi \left(0.18\right)}\right)$	$\left(0.27e^{2i\pi \left(0.89\right)},0.45e^{2i\pi \left(0.61\right)},0.45e^{2i\pi \left(0.24\right)}\right)$
${Ǽ}_{3}$	$\left(0.45e^{2i\pi \left(0.61\right)},0.66e^{2i\pi \left(0.28\right)},0.55e^{2i\pi \left(0.25\right)}\right)$	$\left(0.59e^{2i\pi \left(0.45\right)},0.62e^{2i\pi \left(0.45\right)},0.17e^{2i\pi \left(0.61\right)}\right)$
${Ǽ}_{4}$	$\left(0.36e^{2i\pi \left(0.58\right)},0.46e^{2i\pi \left(0.67\right)},0.07e^{2i\pi \left(0.32\right)}\right)$	$\left(0.36e^{2i\pi \left(0.68\right)},0.36e^{2i\pi \left(0.56\right)},0.28e^{2i\pi \left(0.19\right)}\right)$
${Ǽ}_{5}$	$\left(0.48e^{2i\pi \left(0.67\right)},0.19e^{2i\pi \left(0.37\right)},0.14e^{2i\pi \left(0.25\right)}\right)$	$\left(0.56e^{2i\pi \left(0.67\right)},0.19e^{2i\pi \left(0.37\right)},0.55e^{2i\pi \left(0.55\right)}\right)$
	$J_{3}$	$J_{4}$
${Ǽ}_{1}$	$\left(0.66e^{2i\pi \left(0.27\right)},0.43e^{2i\pi \left(0.61\right)},0.13e^{2i\pi \left(0.22\right)}\right)$	$\left(0.56e^{2i\pi \left(0.55\right)},0.23e^{2i\pi \left(0.09\right)},0.45e^{2i\pi \left(0.78\right)}\right)$
${Ǽ}_{2}$	$\left(0.35e^{2i\pi \left(0.36\right)},0.35e^{2i\pi \left(0.55\right)},0.34e^{2i\pi \left(0.26\right)}\right)$	$\left(0.19e^{2i\pi \left(0.46\right)},0.53e^{2i\pi \left(0.47\right)},0.18e^{2i\pi \left(0.67\right)}\right)$
${Ǽ}_{3}$	$\left(0.56e^{2i\pi \left(0.74\right)},0.18e^{2i\pi \left(0.32\right)},0.27e^{2i\pi \left(0.41\right)}\right)$	$\left(0.49e^{2i\pi \left(0.47\right)},0.29e^{2i\pi \left(0.38\right)},0.76e^{2i\pi \left(0.45\right)}\right)$
${Ǽ}_{4}$	$\left(0.37e^{2i\pi \left(0.52\right)},0.42e^{2i\pi \left(0.38\right)},0.55e^{2i\pi \left(0.27\right)}\right)$	$\left(0.55e^{2i\pi \left(0.48\right)},0.29e^{2i\pi \left(0.45\right)},0.67e^{2i\pi \left(0.38\right)}\right)$
${Ǽ}_{5}$	$\left(0.51e^{2i\pi \left(0.59\right)},0.38e^{2i\pi \left(0.81\right)},0.09e^{2i\pi \left(0.33\right)}\right)$	$\left(0.47e^{2i\pi \left(0.57\right)},0.47e^{2i\pi \left(0.48\right)},0.19e^{2i\pi \left(0.55\right)}\right)$

**Table 3:** Contains the results of the CSFAAWA operator at Ⱥ.

	CSFAAWA
${Ǽ}_{1}$	$\left(0.4056e^{2i\pi \left(0.3388\right)},0.6422e^{2i\pi \left(0.4427\right)},0.4934e^{2i\pi \left(0.6376\right)}\right)$
${Ǽ}_{2}$	$\left(0.2569e^{2i\pi \left(0.5974\right)},0.5731e^{2i\pi \left(0.6626\right)},0.6049e^{2i\pi \left(0.5466\right)}\right)$
${Ǽ}_{3}$	$\left(0.3769e^{2i\pi \left(0.4660\right)},0.5819e^{2i\pi \left(0.6274\right)},0.5671e^{2i\pi \left(0.6516\right)}\right)$
${Ǽ}_{4}$	$\left(0.3062e^{2i\pi \left(0.4229\right)},0.6505e^{2i\pi \left(0.7185\right)},0.4768e^{2i\pi \left(0.5577\right)}\right)$
${Ǽ}_{5}$	$\left(0.3550e^{2i\pi \left(0.4500\right)},0.5484e^{2i\pi \left(0.6971\right)},0.4373e^{2i\pi \left(0.6368\right)}\right)$

**Table 4:** Contains the results of the CSFAAWG operator at Ⱥ.

	CSFAAWG
${Ǽ}_{1}$	$\left(0.7164e^{2i\pi \left(0.6080\right)},0.2929e^{2i\pi \left(0.4332\right)},0.2974e^{2i\pi \left(0.4822\right)}\right)$
${Ǽ}_{2}$	$\left(0.5798e^{2i\pi \left(0.6767\right)},0.3033e^{2i\pi \left(0.3775\right)},0.3493e^{2i\pi \left(0.3750\right)}\right)$
${Ǽ}_{3}$	$\left(0.7507e^{2i\pi \left(0.7580\right)},0.4083e^{2i\pi \left(0.2620\right)},0.4488e^{2i\pi \left(0.3579\right)}\right)$
${Ǽ}_{4}$	$\left(0.6605e^{2i\pi \left(0.7707\right)},0.2765e^{2i\pi \left(0.4017\right)},0.3918e^{2i\pi \left(0.2121\right)}\right)$
${Ǽ}_{5}$	$\left(0.7427e^{2i\pi \left(0.8113\right)},0.2609e^{2i\pi \left(0.5176\right)},0.3102e^{2i\pi \left(0.3397\right)}\right)$

Step 3: To determine a more accurate alternative, investigate score values by using both consequences of the CSFAAWA and CSFAAWG operators. All computed results of the score values are drawn in (Table 5).

**Table 5:** Score values obtained by the CSFAAWA and CSFAAWG operators.

	$S\left({Ǽ}_{1}\right)$	$S\left({Ǽ}_{2}\right)$	$S\left({Ǽ}_{3}\right)$	$S\left({Ǽ}_{4}\right)$	$S\left({Ǽ}_{5}\right)$	Ranking and ordering
CSFAAWA	0.5035	0.4985	0.4802	0.4658	0.4908	${Ǽ}_{1}≻{Ǽ}_{2}≻{Ǽ}_{5}≻{Ǽ}_{3}≻{Ǽ}_{4}$
CSFAAWG	0.7147	0.7162	0.7622	0.7657	0.7770	${Ǽ}_{5}≻{Ǽ}_{4}≻{Ǽ}_{3}≻{Ǽ}_{2}≻{Ǽ}_{1}$

**Fig. 1:**
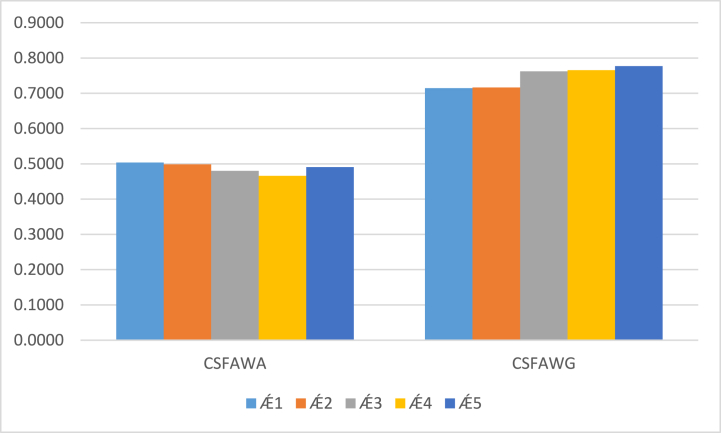
Score values of the CSFAAWA and CSFAAWG operators.

Step 4: From the computed results of the score values, ${Ǽ}_{1}$ and ${Ǽ}_{5}$ are more appropriate electric motor cars, which fulfil defined criteria by the experts. All these investigated score values by the CSFAAWA and CSFAAWG operators are plotted in Fig. 1.

### 6.3 Influence study

By the setting of different parametric values of Ⱥ in our proposed methodologies of the CSFAAWA and CSFAAWAG operators. We studied the impact on the results of our current approaches by considering different parametric values. All investigated results from the CSFAAWA and CSFAAWG operators are listed in (Table 6) and (Table 7). From (Table 6), we examined the results of the CSFAAWA operator are gradually maximized by increasing the parametric values of Ⱥ and raking of score values at Ⱥ≥10 remain unchanged ${Ǽ}_{2}≻{Ǽ}_{1}≻{Ǽ}_{3}≻{Ǽ}_{5}≻{Ǽ}_{4}$.

**Table 6:** Contains results obtained by the CSFAAWA operator for different values of Ⱥ.

	$S\left({Ǽ}_{1}\right)$	$S\left({Ǽ}_{2}\right)$	$S\left({Ǽ}_{3}\right)$	$S\left({Ǽ}_{4}\right)$	$S\left({Ǽ}_{5}\right)$	Ranking and ordering
Ⱥ=1	0.4426	0.4361	0.4373	0.4315	0.4613	${Ǽ}_{5}≻{Ǽ}_{1}≻{Ǽ}_{3}≻{Ǽ}_{2}≻{Ǽ}_{4}$
Ⱥ=3	0.5035	0.4985	0.4802	0.4658	0.4908	${Ǽ}_{1}≻{Ǽ}_{2}≻{Ǽ}_{5}≻{Ǽ}_{3}≻{Ǽ}_{4}$
Ⱥ=10	0.5572	0.5673	0.5285	0.5097	0.5237	${Ǽ}_{2}≻{Ǽ}_{1}≻{Ǽ}_{3}≻{Ǽ}_{5}≻{Ǽ}_{4}$
Ⱥ=50	0.5810	0.6012	0.5570	0.5378	0.5440	${Ǽ}_{2}≻{Ǽ}_{1}≻{Ǽ}_{3}≻{Ǽ}_{5}≻{Ǽ}_{4}$
Ⱥ=85	0.5836	0.6048	0.5603	0.5410	0.5463	${Ǽ}_{2}≻{Ǽ}_{1}≻{Ǽ}_{3}≻{Ǽ}_{5}≻{Ǽ}_{4}$
Ⱥ=100	0.5842	0.6056	0.5610	0.5416	0.5468	${Ǽ}_{2}≻{Ǽ}_{1}≻{Ǽ}_{3}≻{Ǽ}_{5}≻{Ǽ}_{4}$
Ⱥ=140	0.5851	0.6068	0.5621	0.5427	0.5476	${Ǽ}_{2}≻{Ǽ}_{3}≻{Ǽ}_{1}≻{Ǽ}_{5}≻{Ǽ}_{4}$
Ⱥ=165	0.5854	0.6073	0.5626	0.5431	0.5479	${Ǽ}_{2}≻{Ǽ}_{3}≻{Ǽ}_{1}≻{Ǽ}_{5}≻{Ǽ}_{4}$
Ⱥ=190	0.5857	0.6076	0.5629	0.5434	0.5481	${Ǽ}_{2}≻{Ǽ}_{3}≻{Ǽ}_{1}≻{Ǽ}_{5}≻{Ǽ}_{4}$
Ⱥ=215	0.5858	0.6079	0.5631	0.5437	0.5483	${Ǽ}_{2}≻{Ǽ}_{3}≻{Ǽ}_{1}≻{Ǽ}_{5}≻{Ǽ}_{4}$
Ⱥ=250	0.5861	0.6082	0.5634	0.5439	0.5485	${Ǽ}_{2}≻{Ǽ}_{3}≻{Ǽ}_{1}≻{Ǽ}_{5}≻{Ǽ}_{4}$

**Table 7:** Contains results obtained by the CSFAAWG operator for different values of Ⱥ.

	$S\left({Ǽ}_{1}\right)$	$S\left({Ǽ}_{2}\right)$	$S\left({Ǽ}_{3}\right)$	$S\left({Ǽ}_{4}\right)$	$S\left({Ǽ}_{5}\right)$	Ranking and ordering
Ⱥ=1	0.7549	0.7553	0.7978	0.7849	0.8094	${Ǽ}_{5}≻{Ǽ}_{3}≻{Ǽ}_{4}≻{Ǽ}_{2}≻{Ǽ}_{1}$
Ⱥ=3	0.7147	0.7162	0.7622	0.7657	0.7770	${Ǽ}_{5}≻{Ǽ}_{4}≻{Ǽ}_{3}≻{Ǽ}_{2}≻{Ǽ}_{1}$
Ⱥ=10	0.6746	0.6783	0.7262	0.7416	0.7495	${Ǽ}_{5}≻{Ǽ}_{4}≻{Ǽ}_{3}≻{Ǽ}_{2}≻{Ǽ}_{1}$
Ⱥ=50	0.6508	0.6553	0.7050	0.7254	0.7339	${Ǽ}_{5}≻{Ǽ}_{4}≻{Ǽ}_{3}≻{Ǽ}_{2}≻{Ǽ}_{1}$
Ⱥ=85	0.6480	0.6525	0.7024	0.7233	0.7317	${Ǽ}_{5}≻{Ǽ}_{4}≻{Ǽ}_{3}≻{Ǽ}_{2}≻{Ǽ}_{1}$
Ⱥ=100	0.6473	0.6519	0.7018	0.7228	0.7312	${Ǽ}_{5}≻{Ǽ}_{4}≻{Ǽ}_{3}≻{Ǽ}_{2}≻{Ǽ}_{1}$
Ⱥ=140	0.6463	0.6509	0.7008	0.7221	0.7304	${Ǽ}_{5}≻{Ǽ}_{4}≻{Ǽ}_{3}≻{Ǽ}_{2}≻{Ǽ}_{1}$
Ⱥ=165	0.6460	0.6505	0.7005	0.7218	0.7301	${Ǽ}_{5}≻{Ǽ}_{4}≻{Ǽ}_{3}≻{Ǽ}_{2}≻{Ǽ}_{1}$
Ⱥ=190	0.6457	0.6503	0.7002	07216	0.7298	${Ǽ}_{5}≻{Ǽ}_{4}≻{Ǽ}_{3}≻{Ǽ}_{2}≻{Ǽ}_{1}$
Ⱥ=215	0.6455	0.6500	0.7000	0.7214	0.7296	${Ǽ}_{5}≻{Ǽ}_{4}≻{Ǽ}_{3}≻{Ǽ}_{2}≻{Ǽ}_{1}$
Ⱥ=250	0.6452	0.6498	0.6998	0.7212	0.7294	${Ǽ}_{5}≻{Ǽ}_{4}≻{Ǽ}_{3}≻{Ǽ}_{2}≻{Ǽ}_{1}$

**Fig. 2:**
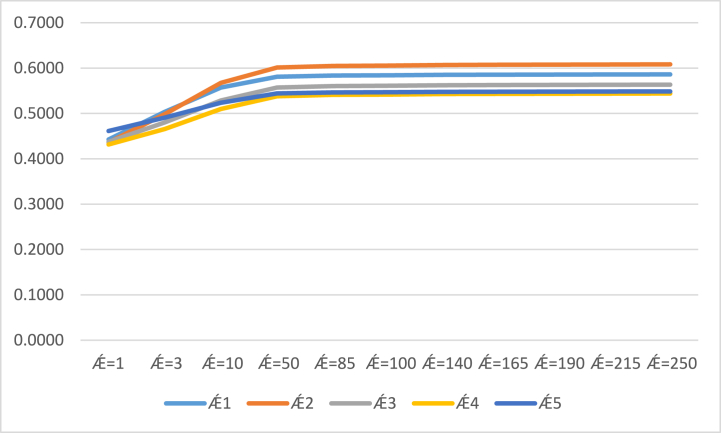
Results of the CSFAAWA operator by the variation of Ⱥ.

**Fig. 3:**
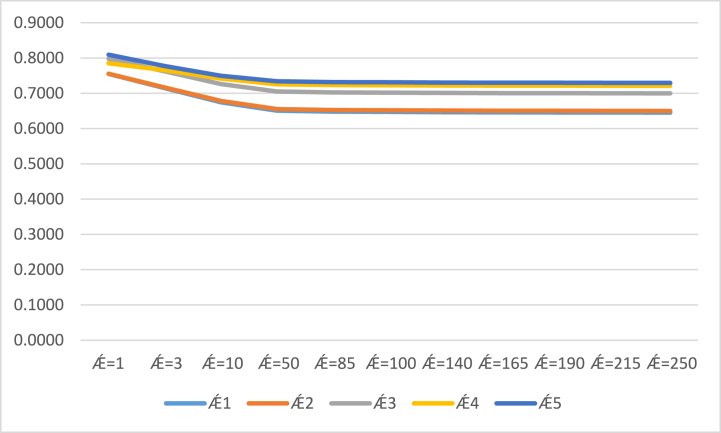
Results of the CSFAAWG operator by the variation of Ⱥ.

Similarly, from (Table 7), by observing the results of the score values which are obtained from the CSFAAWG operator, its clear results of the score values gradually decrease by increasing the parametric values of Ⱥ in the CSFAAWG operator and the ranking of score values still unchanged ${Ǽ}_{5}≻{Ǽ}_{3}≻{Ǽ}_{4}≻{Ǽ}_{2}≻{Ǽ}_{1}$ at all values of Ⱥ. We also explored the results of score values, which are listed in (Table 6) and (Table 7) graphically in Fig. 2 and Fig. 3 respectively.

## 7 Comparative study

**Fig. 4:**
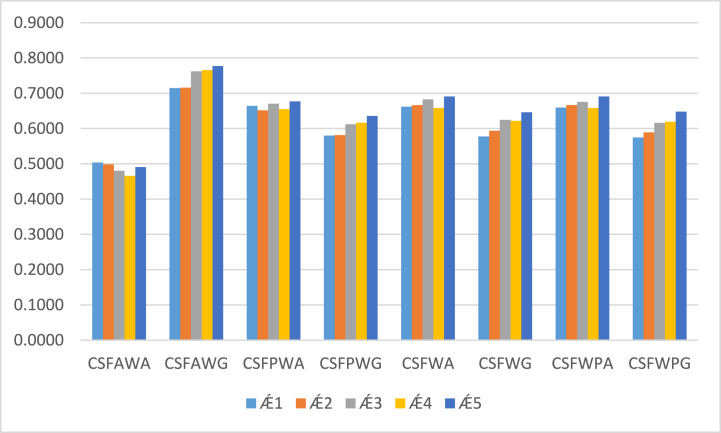
Results of the existing methodologies.

To verify the compatibility and reliability of our discussed approaches, including CSFAWA and CSFAWG operators. We applied some existing approaches [53,55] to evaluate different alternatives based on assigning degrees of attributes drawn in the decision matrix (Table 2). The CSF prioritized weighted AOs established by Akram et al. [53], CSF average and geometric operators based on some prominent degrees of attributes also introduced by Akram et al. [54] and CSF average and geometric operators based on power-weighted aggregation tools were presented by Naeem et al. [55]. After computing and investigating CSF information by utilizing the discussed decision-making algorithm, the result of the score values and their ranking are listed in (Table 8) and Fig. 4.

**Table 8:** Contains the results of the comparative analysis.

Aggregation Operators	Score values	Ranking and ordering
CSFAWA	$S\left({Ǽ}_{1}\right)=0.5035,S\left({Ǽ}_{2}\right)=0.4985\text{,}$ $S\left({Ǽ}_{3}\right)=0.4802,S\left({Ǽ}_{4}\right)=0.4658$ $S\left({Ǽ}_{5}\right)=0.4908$	${Ǽ}_{1}≻{Ǽ}_{2}≻{Ǽ}_{5}≻{Ǽ}_{3}≻{Ǽ}_{4}$
CSFAWG	$S\left({Ǽ}_{1}\right)=0.7147,S\left({Ǽ}_{2}\right)=0.7162$ $S\left({Ǽ}_{3}\right)=0.7622,S\left({Ǽ}_{4}\right)=0.7657$ $S\left({Ǽ}_{5}\right)=0.7770$	${Ǽ}_{5}≻{Ǽ}_{4}≻{Ǽ}_{3}≻{Ǽ}_{2}≻{Ǽ}_{1}$
CSFPWA by Akram et al. [53]	$S\left({Ǽ}_{1}\right)=0.6645,S\left({Ǽ}_{2}\right)=0.6514$ $S\left({Ǽ}_{3}\right)=0.6706,S\left({Ǽ}_{4}\right)=0.6551$ $S\left({Ǽ}_{5}\right)=0.6768$	${Ǽ}_{5}≻{Ǽ}_{3}≻{Ǽ}_{1}≻{Ǽ}_{4}≻{Ǽ}_{2}$
CSFPWG by Akram et al. [53]	$S\left({Ǽ}_{1}\right)=0.5800,S\left({Ǽ}_{2}\right)=0.5813$ $S\left({Ǽ}_{3}\right)=0.6119,S\left({Ǽ}_{4}\right)=0.6165$ $S\left({Ǽ}_{5}\right)=0.6358$	${Ǽ}_{5}≻{Ǽ}_{4}≻{Ǽ}_{3}≻{Ǽ}_{2}≻{Ǽ}_{1}$
CSFWA by Akram et al. [54]	$S\left({Ǽ}_{1}\right)=0.6620,S\left({Ǽ}_{2}\right)=0.6664$ $S\left({Ǽ}_{3}\right)=0.6828,S\left({Ǽ}_{4}\right)=0.6582$ $S\left({Ǽ}_{5}\right)=0.6908$	${Ǽ}_{5}≻{Ǽ}_{3}≻{Ǽ}_{2}≻{Ǽ}_{1}≻{Ǽ}_{4}$
CSFWG by Akram et al. [54]	$S\left({Ǽ}_{1}\right)=0.5775,S\left({Ǽ}_{2}\right)=0.5937$ $S\left({Ǽ}_{3}\right)=0.6246,S\left({Ǽ}_{4}\right)=0.6220$ $S\left({Ǽ}_{5}\right)=0.6461$	${Ǽ}_{5}≻{Ǽ}_{3}≻{Ǽ}_{4}≻{Ǽ}_{1}≻{Ǽ}_{2}$
CSFWPA by Naeem et al. [55]	$S\left({Ǽ}_{1}\right)=0.6597,S\left({Ǽ}_{2}\right)=0.6666$ $S\left({Ǽ}_{3}\right)=0.6755,S\left({Ǽ}_{4}\right)=0.6583$ $S\left({Ǽ}_{5}\right)=0.6909$	${Ǽ}_{5}≻{Ǽ}_{3}≻{Ǽ}_{2}≻{Ǽ}_{1}≻{Ǽ}_{4}$
CSFWPG by Naeem et al. [55]	$S\left({Ǽ}_{1}\right)=0.5748,S\left({Ǽ}_{2}\right)=0.5891$ $S\left({Ǽ}_{3}\right)=0.6162,S\left({Ǽ}_{4}\right)=0.6191$ $S\left({Ǽ}_{5}\right)=0.6480$	${Ǽ}_{5}≻{Ǽ}_{4}≻{Ǽ}_{3}≻{Ǽ}_{1}≻{Ǽ}_{2}$

## 8 Conclusion

An area of applied science called operations research effectively uses advanced analytic strategies to enhance decision-making. Operations research includes a technique called the MCDM assessment that you can use to compare several possibilities that may be in conflict and decide which is more appropriate. Therefore, the MCDM technique plays an essential role in every field of the decision-making process. In this article, we exposed the theory of CSFSs and their basic characteristics. A CSFS contains extensive information about any object with four terms of membership values. In CSFS, each term has two aspects in the form of amplitude and phase terms. Some necessary operations of the Aczel-Alsina aggregation tools are also characterized under the system of CSF information. Some robust aggregation approaches are characterized by a system of CSF information, including CSFAAWA and CSFAAWG operators. We also study the appropriate characteristics and special cases of our developed methodologies. An experimental case is also illustrated to evaluate a suitable electric motor car based on the MCDM technique. To verify the applicability of the currently discussed approaches, we demonstrate a comparison method to compare the results of previous approaches with the currently proposed AOs.

Sometimes our developed strategies must be evaluated given information in the form of CSF, and experts cannot reach a desirable and appropriate optimal option or solution. To awkward such situations, we have to extend our proposed research in the framework of T-spherical fuzzy sets and the complex T-spherical fuzzy circumstances theory. Next, we will apply our invented approaches to resolve different real-life applications, including game theory, computation and web development, artificial intelligence, waste management, pattern recognition, logistic operators, social sciences and networking. These future developments would allow to contribute to the solution of complex real-life problems such as decision making with multiple stakeholders related to urban mobility governance. Keeping in mind the significance of our invented approaches and Aczel-Alsina aggregation tools, we will explore our presented methodologies in the field of T-spherical FSs [56] and bipolar soft sets [57] and complex bipolar soft sets [58]. Some advanced decision-making of the Three-Way Multiattribute Decision-Making techniques [59] will also study under our presented strategies.

## Funding

This article was partially funded by the 10.13039/501100000780European Commission through the SENATOR project (H2020MG-2018-2020, RIA, project n. 861,540).

## Author contribution statement

Sarbast Moslem, Abrar Hussain, Kifayat Ullah, Tapan Senapati: Conceived and designed the experiments; Performed the experiments; Analyzed and interpreted the data; Contributed reagents, materials, analysis tools or data; Wrote the paper. </p>

## Data availability statement

Data will be made available on request.

## Declaration of competing interest

The authors declare that they have no known competing financial interests or personal relationships that could have appeared to influence the work reported in this paper.
